# Impact of Nutrition on Age-Related Epigenetic RNA Modifications in Rats

**DOI:** 10.3390/nu14061232

**Published:** 2022-03-15

**Authors:** Patrizia D’Aquila, Francesco De Rango, Ersilia Paparazzo, Maurizio Mandalà, Dina Bellizzi, Giuseppe Passarino

**Affiliations:** Department of Biology, Ecology and Earth Science, University of Calabria, 87036 Rende, Italy; patrizia.daquila@unical.it (P.D.); francesco.derango@unical.it (F.D.R.); ersilia.paparazzo@unical.it (E.P.); m.mandala@unical.it (M.M.); giuseppe.passarino@unical.it (G.P.)

**Keywords:** aging, tissue-specificity, RNA methylation, low-calorie diet, nutrition, writer, eraser and reader enzymes, biomarkers

## Abstract

Nutrition plastically modulates the epigenetic landscape in various tissues of an organism during life via epigenetic changes. In the present study, to clarify whether this modulation involves RNA methylation, we evaluated global RNA methylation profiles and the expression of writer, reader, and eraser genes, encoding for enzymes involved in the RNA methylation. The study was carried out in the heart, liver, and kidney samples from rats of different ages in response to a low-calorie diet. We found that, although each tissue showed peculiar RNA methylation levels, a general increase in these levels was observed throughout the lifespan as well as in response to the six-month diet. Similarly, a prominent remodeling of the expression of writer, reader, and eraser genes emerged. Our data provide a comprehensive overview of the role exerted by diet on the tissue-specific epigenetic plasticity of RNA according to aging in rats, providing the first evidence that methylation of RNA, similarly to DNA methylation, can represent an effective biomarker of aging. What is more, the fact that it is regulated by nutrition provides the basis for the development of targeted approaches capable of guaranteeing the maintenance of a state of good health.

## 1. Introduction

Over the last few years, a variety of epigenetic signs has been discovered to modify dynamically every type of RNA molecule [1,2,3,4]. Methylated RNA ImmunoPrecipitation and Sequencing (MeRIP-Seq) studies revealed that the methylation of the N6 site of adenosine (m6A) represents the most prevalent epigenetic modification along with mammalian RNAs, accounting for 0.1–0.4% of adenines. They are preferentially clustered in the coding sequence, around the stop codons, and in the 5′ and 3′ untranslated regions (UTRs) within the conserved consensus motif DRA*CH (where A* denotes the methylable adenosine, D denotes G/A/U; R denotes G/A; H denotes A/C/U) [5,6,7,8,9]. m6A profiles are established by the concerted activity of “writers” and “erasers”, enzymes that add and delete methyl groups from RNAs, respectively. Then, the epigenetic information is decoded by “readers”, namely m6A binding proteins, which translate the m6A profiles in molecular signals involved in a variety of post-transcriptional gene regulation processes, including RNA splicing, translation efficiency, stability/decay, nuclear export, and RNA-protein interaction [10,11,12,13,14,15].

The activity of enzymes involved in m6A reactions is strictly regulated by various substrates, allosteric regulators, and by-products of cellular metabolic pathways. In particular, the one-carbon metabolism is one of the most direct pathways involved in the regulation of m6A methylation. Indeed, the S-adenosylmethionine (AdoMet or SAM), the universal methyl donor group, activates the methyltransferase 3 enzyme (Mettl3) meanwhile the S-adenosyl-L-homocysteine (SAH), a bioproduct of methylation reactions, allosterically inhibits it [16]. On the other hand, kinetic studies revealed that the m6A demethylation, by the erasers alpha-ketoglutarate dependent dioxygenase (Fto) and alkB homolog 5, RNA demethylase (Alkbh5) enzymes, require alpha-ketoglutarate (AKG), oxygen and iron, and is inhibited by succinate and fumarate, all metabolites of the tricarboxylic acid cycle [17,18,19,20]. The direct link between metabolism and RNA m6A demethylation emerges also by the recent study from Wang and coll., which, by testing in vitro a few metabolites, demonstrated that the nicotinamide adenine dinucleotide phosphate (NADP) binds and enhancers Fto demethylation activity [21]. The contribution of NADP on the Fto-dependent m6A demethylation was further corroborated by in vivo studies carried out in cultured cell models of adipogenesis as well as in Fto knockout mice [21].

Furthermore, m6A profiles are correlated to a variety of diseases, including infertility, virus infections, cancer, and metabolic diseases [22,23,24,25]. A dynamic correlation between Fto-dependent m6A and glucose levels was recently identified in Type 2 diabetic subjects as well as a reduction of m6A in key β-cell genes was proposed as a contributor to the pathophysiology of the disease [26,27,28]. At the same time, involvement of m6A in the regulation of lipid metabolism and adipogenesis has been progressively reported. In vivo and in vitro experiments have demonstrated that m6A levels negatively correlate with fat deposition and cell cycle progression and differentiation of preadipocytes [29,30,31,32]. Studies on a mouse model of Non-alcoholic fatty liver disease (NAFLD) revealed that betaine supplementation during adolescence rectified the decreased m6A levels and the increased *Fto* gene expression in response to a high-fat diet, by decreasing de novo lipogenesis and increasing lipolysis, and thus protecting mice from high-fat-induced lipid accumulation in the liver [33]. What is more, knockdown experiments of the m6A methyltransferases inhibit adipocyte differentiation and alter adipogenesis, decrease the serum levels of circulating lipids, as well as affect the circadian clock in mice [34,35]. Lastly, a variety of dietary regimens is demonstrating the correlation between nutritional metabolism and RNA methylation profiles in several tissues. Kaspi and coll. (2018) revealed that low-fat diet during pregnancy inversely correlates to the hypothalamic expression levels of *Mettl3* and *Fto* genes and thus to the m6A content of the offspring [36]. What is more, maternal high-fat intake affects mRNA m6A modifications and its related genes in the visceral fat of three-weeks-old offspring [37]. The dietary supplementation of betaine, widely recognized as global methylation regulator, counteracts the impairment of adipose tissue function induced by high-fat diet in mice through the improvement of mitochondrial function and the regulation of m6A levels [38]. It has been recently demonstrated that the supplementation of dietary polyphenols, including curcumin and resveratrol, exerts many health-protective effects strictly through the m6A RNA methylation [39,40].

Therefore, given that the m6A profiles, on the one hand, are involved in a variety of metabolic signaling pathways and, on the other, are influenced by the nutritional physiology and metabolism, we explored whether changes in the global RNA methylation status occur in rats during aging and according to changes in eating habit. What is more, the expression profiles of genes encoding for writer (*Mettl3*, *Mettl14*, *Mettl16*, *Rbm15*, *Virma*, *Wtap*, *Zc3h13*), reader (*Hnrnpa2b1*, *Hnrnpc*, *Igf2bp1*, *Igf2bp2*, *Igf2bp3*, *Ythdc1*, *Ythdc2*, *Ythdf1*, *Ythdf2*, and *Ythdf3*), and eraser (*Alkbh5* and *Fto*) enzymes have been determined.

## 2. Materials and Methods

### 2.1. Study Design

The study was carried out on RNA samples extracted from heart, liver, and kidney tissues from rats of different ages (27, 36, and 96 weeks of age) and fed with standard and low-calorie diet for 24 weeks. The RNA samples were both used for ELISA assays to quantify the global methylation levels and retrotranscribed to evaluate, by comparative-relative qPCR, the expression levels of the genes involved in the RNA methylation process. Description and functional annotation of the analyzed genes, acquired from Rat Genome Database (RGD), was reported in Tables S1 (writers), Tables S2 (readers), and Tables S3 (erasers).

### 2.2. Animals

Experiments were performed on Sprague–Dawley rats breeding locally in the animal care facility of the University of Calabria (Italy). Animals (*n* = 3 for each experimental condition) were housed in light- (12:12 h light–dark cycle) and temperature- (22 °C) controlled rooms with free access to food (ssniff diet V1535, Ferdinand–Gabriel–Weg 16 D-59494 Soest, German, metabolizable energy 3.057 kcal/kg) and water. Rats of 3, 12, and 72 weeks of age were divided into two groups: The first (control group) was fed with a standard diet, the second (treated group) was fed with low-calorie diet (60% of the intake) for a total period of 24 weeks, thus obtaining rats of 27, 36, and 96 weeks of ages.

Water and food intake were recorded every other day while body mass was recorded monthly. Animals were euthanized with isoflurane followed by cervical transection and immediately the organs (heart, kidney, and liver) were removed and frozen at −80 °C. All procedures were conducted in accordance with the European Guidelines for the care and use of laboratory animals (Directive 2010/63/EU) and in accordance with Italian law; the Ethical committee of the Ministry of Health authorized the study with authorization number 295/2016-PR.

### 2.3. RNA Extraction

Fifty milligrams of frozen heart, kidney, and liver were excised and homogenized in buffer RTL, total RNA was purified using RNeasy Mini Kit (Qiagen, Milan, Italy) in accordance with the manufacturer’s recommendations, and RNA samples were treated with DNA-free DNase to remove any residual genomic DNA contamination. The ratio of the absorbance at 260 and 280 nm was used to assess the RNA purity of RNA preparations.

### 2.4. Quantification of N6-Methyladenosine RNA Levels

The global RNA methylation levels of 6-methyladenosine were quantified by the m6A RNA methylation quantification kit (Epigentek, Farmingdale, NY, USA). Two hundred nanograms RNAs were coated on per assay well, and the m6A content was captured and detected according to the manufacturer’s instructions.

Shortly, the methylated fraction of total RNA, through ELISA-like reactions, was recognized by the m6A antibody and quantified in a microplate spectrophotometer by reading the absorbance at 450 nm.

In each experiment, the percentage of m6A was calculated using the second-order regression equation of a standard curve that was constructed by mixing equivalent molar concentrations at different ratios of full unmethylated and methylated control DNA. Each sample was analyzed in triplicate.

### 2.5. Expression Profile Analysis of Enzymes Involved in RNA Methylation

The reverse-transcriptase-polymerase chain reactions (RT-PCR) were carried out by using the ImPromII Kit (Promega, Milan, Italy). First, an RT mix including 500 ng of total RNA and 0.5 μg of oligo-dT primers were pre-heated at 70 °C for 5 min. Then, the reaction was carried out in a 40 μL final volume containing 1X RT buffer, 0.5 mM of each dNTP, 3 mM of MgCl2, 20 U of RNase inhibitor, and 5 U of reverse transcriptase. The mix was incubated at 25 °C for 5 min, then 37 °C for 1 h and, successively, at 95 °C for 10 min to inactivate the reverse transcriptase.

The cDNAs obtained were then used as a template for real-time PCRs carried out in a QuantStudio3 real-time PCR system (Thermo Fisher Scientific, Monza, Italy). Forward and reverse primers are reported in Table S4.

The final PCR mixture (15 µL) contained 1 µL of cDNA, SensiFAST SYBR Hi-ROX Mix 1X (Bioline, London, UK) and 0.2 µM of each primer. The thermal profile used for the reaction included a 2-min heat activation of the enzyme at 95 °C, followed by 35 cycles of denaturation at 95 °C for 15 s and annealing/extension at optimal temperature for each primer pair (Table S1) for 60 s, followed by melt analysis ramping at 60–95 °C. All measurements were taken in the log phase of amplification. Negative controls (in which water instead of cDNA was added) were also run in each plate. Real-time PCR data were analyzed by the comparative CT method, according to which the 2^dCt values of each target gene were normalized to that of the housekeeping gene Glyceraldehyde-3-phosphate dehydrogenase (*Gapdh*). Then, the relative quantification of gene expression was determined by using the values of the 27 weeks of age samples as reference values.

### 2.6. Statistical Analysis

All statistical analysis was performed using R Statistical Software (version 4.1.2) [41]. Comparisons between groups were analyzed by an unpaired *t*-test including Welch’s correction. Pearson’s correlations were used to evaluate the association between methylation levels and age. Normality was checked for all data before analysis. After finding the differences in the expression of all genes, we set the absolute value of a log_2_FC (fold change) > 1 and *p*-values < 0.05 as filter conditions to select significantly up- and downregulated expressed genes. Fold change is a measure describing how much a quantity changes between an original and a subsequent measurement. It is defined as the ratio between the two quantities. In bioinformatics, log-ratios are often used for analysis and visualization of fold changes. The logarithm to base 2 is most used, as it is easy to interpret, e.g., a doubling in the original scaling is equal to a log_2_ fold change of 1, a quadrupling is equal to a log_2_ fold change of 2 and so on [42].

As multiple tests were performed, the *p*-values of the results were corrected using the Bonferroni method. The volcano plots graphs used to display the results of the differential expression analyses were created by the enhanced Volcano R package [43].

## 3. Results

### 3.1. Aging and Dietary Effects on Global RNA Methylation Levels

**Figure 1:**
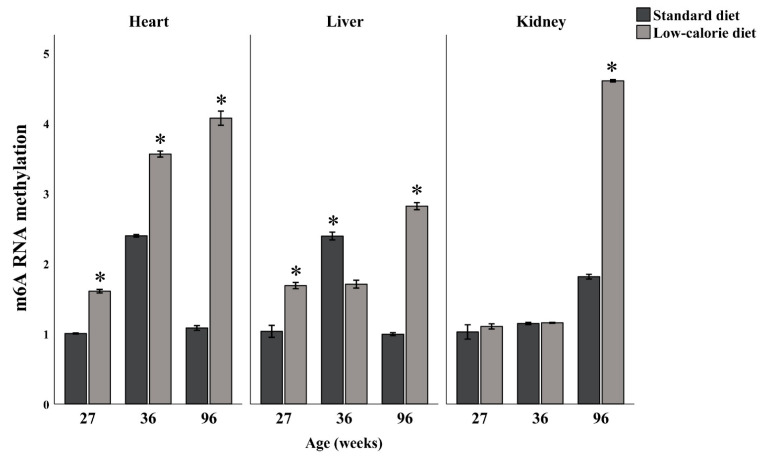
Global RNA methylation levels in the heart, liver, and kidney from rats of 27, 36, and 96 weeks of age, fed with standard and low-calorie diets. The values represent the mean of three independent triplicate experiments with standard deviation. * *p* < 0.001 (Welch’s test Bonferroni adjusted).

The global levels of m6A residues were evaluated in total RNA samples extracted from the heart, liver, and kidney of differently aged rats (27, 36, and 96 weeks of age) fed with standard or six months low-calorie diet, by using an ELISA assay. In Figure 1, for each tissue, age-associated RNA methylation levels are reported. Under standard fed conditions, the levels of RNA methylation in heart and liver increase over the period of life between 27 and 36 weeks of age, and then decline to levels comparable to those of 27 weeks of age, meanwhile a progressive gain of RNA methylation across life was observable in the kidney. Furthermore, remarkable RNA methylation changes were observed, according to the age and the tissues, following to the six months low-calorie diet. Specifically, in the heart the low-calorie diet induces a generalized increase of the RNA methylation levels compared to the control diet. This increase is also appreciable at 96 weeks of age in the kidney. In the liver, a hyper-methylation of RNA was induced by the low-calorie diet in samples at 27 and 96 weeks of age, while a hypo-methylation at 36 weeks of age compared to the control diet.

A correlation analysis between global RNA methylation levels and age shows a statistically significant correlation only in the kidney. In the low-calorie diet we observed a significant positive correlation between methylation levels and age for all tissues. In fact, as it is shown in Figure 1, while in the standard diet global RNA methylation levels increased until 36 weeks of age and then decreased, in mice with a low-calorie diet they kept increasing also after this age.

### 3.2. mRNA Expression of Enzymes Involved in m6A RNA Methylation during Age

**Figure 2:**
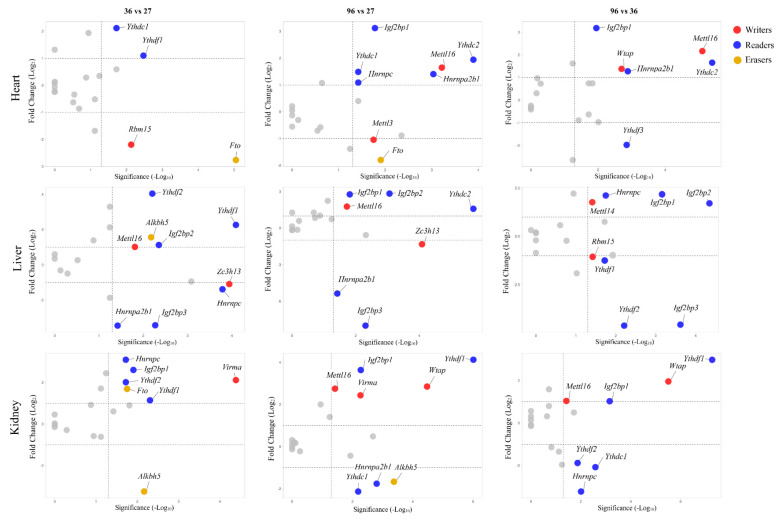
Changes in the expression of writer, reader, and eraser genes in the heart, liver, and kidney from rats during age. The values are reported as log_2_FC (fold change) calculated as the ratio of the mRNA levels between 36 and 27, 96 and 27, and 96 and 36 weeks of age. Scattered points represent all the analyzed genes; red, blue, and yellow dots represent writer, reader, and eraser genes, respectively, that encounter significant differences in levels of expression with age. Grey dots represent unregulated genes. The x-axis represents the Bonferroni *p*-value adjusted in −log_10_ format, the y-axis shows the log_2_ ratio of gene expression levels of one gene at two different ages. The dotted lines represent Bonferroni cut-off (*p*-values < 0.05) and the two-fold change.

The expression profiles of genes encoding for writer, reader, and eraser enzymes involved in m6A RNA methylation were evaluated in the heart, liver, and kidney samples from rats of different ages by quantitative real-time PCR assays (Figure 2 and Figure S1). In the text, only those significant (Bonferroni-adjusted *p*-values < 0.05) results associated with an absolute value of a log_2_FC (fold change) > 1 were reported.

In the heart, the expression of most of the writer genes, including *Mettl16* and *Wtap*, remained constant until 36 weeks of age, then, increased while that of *Rbm15* gene decreased between 27 and 36 weeks of age and then, remained constant. The reader genes underwent a greater modulation of gene expression over the course of age. Increased levels of *Igf2bp1*, *Hnrpc*, *Hnrnpa2b1*, and *Ythdc2* genes were detected from 36 weeks of age onward. Additionally, *Ythdc1* and *Ythdf1* gene expression levels rose between 27 and 36 weeks of age, and then, remained constant. A decline in *Ythdf3* late in life was also observed. The sole eraser gene that showed variations in expression as a function of age was *Fto*, whose expression declined between 27 and 36 weeks of age, and then remained constant.

In the liver, the expression of writer genes over the age showed variable trends; increased expression levels with age were observed for *Mettl16* between 27 and 36 weeks of age. An increase and decrease of *Mettl14* and *Rbm15* levels, respectively, were observed from 36 weeks of age onward. Conversely, *Zc3h13* levels declined between 27 and 36 weeks of age and then remained constant. Regarding the reader genes, a progressive increase in *Igf2bp2* expression occurred with age. Increased expression levels were also reported for *Igf2bp1* gene, from 36 weeks of age onward, and for *Ythdf1* and *Ythdf2* between 27 and 36 weeks of age, followed by a progressive decline. Contrariwise, a progressive decline in gene expression levels with age occurred for *Igf2bp3* and *Hnrnpa2b1* genes, the latter between 27 and 36 weeks of age. The expression of *Alkbh5* eraser genes increased early in life and then remains constant.

In the kidney, the writer genes underwent a general hyper-expression according to age. Indeed, *Wtap* genes expression levels progressively rise with age. Likewise, *Virma* levels increased between 27 and 36 weeks of age, as well as *Mettl16* levels increasing from 36 weeks of age onward. A hyper-expression trend was also observable for most of the reader genes between 27 and 36 weeks of age. Then, both increased (*Igf2bp1* and *Ythdf1*) and decreased (*Hnrnpc* and *Ythdf2*) expression levels were observed from 36 weeks of age onward. Regarding the eraser genes, a decrease in *Alkbh5* and an increase in *Fto* levels were observed between 27 and 36 weeks of age.

### 3.3. Impact of Low-Calorie Diet on mRNA Expression of Enzymes Involved in m6A RNA Methylation

**Figure 3:**
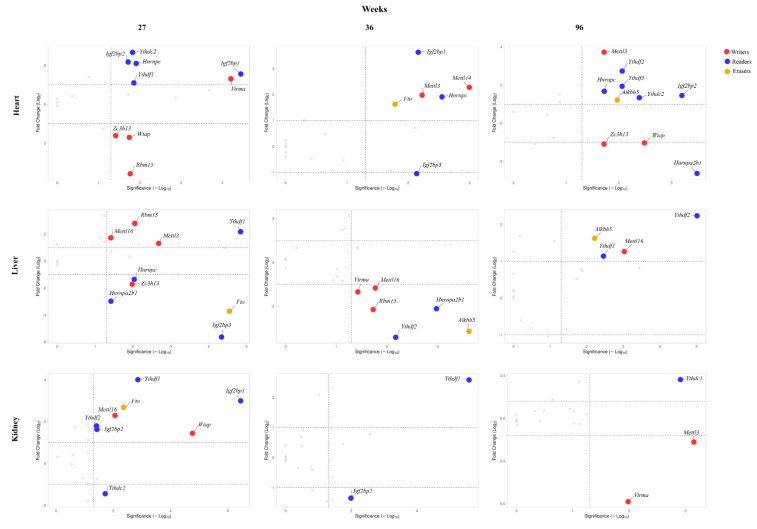
Changes in the expression of writer, reader, and eraser genes in the heart, liver, and kidney from rats according to diet for each analyzed age. The values are reported as log_2_FC (fold change) calculated as the ratio of the mRNA levels between low-calorie and standard diet at 27, 36, and 96 weeks of age. Scattered points represent all the analyzed genes; red, blue, and yellow dots represent writer, reader, and eraser genes, respectively, that encounter significant differences in levels of expression with age. Grey dots represent unregulated genes. The x-axis represents the Bonferroni *p*-value adjusted in −log_10_ format, the y-axis shows log_2_ ratio of gene expression levels between standard and low-calorie diet. The dotted lines represent Bonferroni cut-off (*p*-values < 0.05) and the two-fold change.

To identify any effects exerted by the low-calorie diet on writers, readers, and erasers gene expression, we evaluated, for each age and tissue, the log_2_FC (fold change) of gene expression levels in samples undergoing standard and low-calorie diet (Figure 3 and Figure S1). We found that diet exerts a greater remodeling of gene expression in the heart and liver than in the kidney. In the heart, the effect of diet on writer gene expression resulted in an increase of the levels of *VIRMA* at 27 weeks of age, *Mettl3* and *Mettl14* at 36 weeks of age, and *Mettl3* at 96 weeks of age. On the contrary, the diet induced a decrease of the levels of *Zc3h13*, *Wtap*, and *Rbm15* at 27 weeks of age and of *Zc3h13* and *Wtap* at 96 weeks of age. A reduction in the expression levels was also observed for *Igf2bp3* and *Hnrnpa2b1* genes at 36 and 96 weeks of age, respectively. In addition, we observed, under a low-calorie diet, a significant increase in the expression of numerous reader genes, namely *Igf2bp1*, *Igf2bp2*, *Hnrnpc*, *Ythdf1*, and *Ythdc2* at 27 weeks of age, *Igf2bp1* and *Hnrnpc* at 36 weeks of age, and *Igf2bp2*, *Hnrnpc*, *Ythdc2 Ythdf2*, and *Ythdf3* at 96 weeks of age. As for the eraser genes, a raise of *Fto* and *Alkbh5* gene expression was observed at 36 and 96 weeks of age, respectively.

In the liver, the low-calorie diet induces, on writer genes, a raise in the expression levels of *Mettl13*, *Mettl16*, and *Rbm15* genes at 27 weeks of age and of the sole *Mettl16* gene at 96 weeks of age with respect to the standard diet. As for the readers, we observed an increase of the expression of *Ythdf1* gene at 27 weeks and of *Ythdf1* and *Ythdf2* genes at 96 weeks of age, and a decrease of *Hnrnpc*, *Hnrnpa2b1* and *Igf2bp3* expression at 27 weeks of age and of *Hnrnpa2b1* and *Ythdf2* genes at 36 weeks of age respect to the standard diet. Regarding the eraser genes, a drop in *Fto* and *Alkbh5* gene expression was observed at 27 and 36 weeks of age, respectively.

In the kidney, the diet induces significant variations mainly in the expression levels of the reader genes. In fact, the expression of *Igf2bp1*, *Igf2bp2*, *Ythdf1*, and *Ythdf2* genes increases at 27 weeks of age, and of *Ythdf1* and *Ythdc1* genes at 36 and 96 weeks of age, respectively, meanwhile a decrease was observed for *Ythdc2*, *Igf2bp2* and *Ythdc1* genes at 27, 36, and 96 weeks of age, respectively. In addition, increased expression levels for the *Mettl16* and *Wtap* writer genes and for the *Fto* eraser gene at 27 weeks of age and decreased levels in the expression of *Mettl3* and *Virma* writer genes at 96 weeks of age were also observed in response to diet.

All raw data obtained in the study—that is, the expression levels of writer, reader, and eraser enzymes determined in the heart, liver, and kidney from rats of 27, 36, and 96 weeks of age fed with standard and low-calorie diet—and all statistical comparisons are reported in Tables S5 and S6. 

## 4. Discussion

As previously reported for epigenetic modifications involving DNA, reversible modifications are also present in RNA molecules. Recently, different studies have characterized their functional role in both physiological and pathological conditions especially in RNA stability, mRNA translation, mRNA splicing, cellular differentiation, hematopoiesis, cancer initiation, and progression [2,4,44,45]. Methylation of DNA has been widely related to the aging process in humans and model organisms as well as age-related DNA methylation changes have been resulted tissue specific. This has led to the identification of tissue-specific markers of chronological and biological age. In addition, a series of factors, including nutrition, were demonstrated to exert profound effects on the above changes [46,47,48,49,50]. To our knowledge, this is the first comprehensive study in which aging, tissue-specificity as well as peculiar nutritional regimens are investigated in relation to RNA methylation.

As previously demonstrated for global DNA methylation levels, we found that RNA methylation changes according to age are also characteristic for each tissue [2,4]. A progressive gain of RNA methylation across life was observable in the kidney; meanwhile, in the heart and liver this gain is only observable during adulthood. This is not surprising because the changes are observed in two highly metabolically active tissues especially in a period of growth where more dynamic regulation of gene expression may be required. A proof of this is that methylation levels tend to decrease progressively in late age, reaching levels comparable to those observed at young age. Since an overall protein synthesis decline characterizes aging process in many organisms, our results from the heart and liver seem to suggest as the decrease of RNA methylation late in life contributes to this decline reducing the number of RNA molecules that can be translated into proteins [51]. Surprisingly, in the kidney, the methylation has a different pattern since it continues to increase over the adulthood age. Overall, the age/dependent differences in RNA methylation could be explained by the different metabolic/aging speed of the tissues. The comparison between rats fed with standard and low-calorie diets led us to observe, next to some fluctuations, a generalized increase of RNA methylation. Particularly, while under standard diet global RNA methylation levels increase until 36 weeks of age and then decrease in heart and liver tissues, in mice with low-calorie diet it keeps increasing also after this age. These results provide further evidence that nutrition exerts its effects through epigenetic modifications [52,53,54]. If these modifications have so far been shown to occur mainly on the DNA, our work demonstrates that RNA is also involved. Considering that the targets of the modifications are all the RNA molecules, the effects of nutrition may result in a consistent post-transcriptional modification thus highlighting that these effects are far more complex than originally believed. In this context, accumulating evidence showing the significance of RNA methylation in metabolism and metabolic diseases strengthen the relationship between nutrition and this methylation. It is noteworthy to draw attention to genes such as *Mettl3*, *Mettl4*, *Wtap*, *Ythdf2*, *Fto* and others, variously reported in literature associated with lipid, glucose, fatty acid metabolism as well as in adipogenesis, insulin production, angiogenesis, and T cell homeostasis, which have also been identified in our research as expressed differently during the lifetime and in low-calorie diet conditions [55,56,57].

The great variability observed in the variation of their expression does not allow us to generalize the overall contribution of writers, readers, and erasers in the establishment of the levels of RNA methylation. Each gene analyzed performs such specific functions that even single variations in their methylation levels can produce specific functional feedback at the molecular levels that now cannot be predicted.

Although we are conscious that the sample size is low, the findings of the present study suggest that RNA methylation is modulated by nutrition, as well as DNA methylation previously reported. As calorie restriction is widely known to be correlated with aging deceleration, it is possible to assume that RNA methylation may be a biomarker of biological age as reported for a few DNA loci [58,59]. On the other hand, the different gene expression levels of writers, readers, and erasers suggest that the modulation of RNA methylation is likely to be quite complex with different genes responding differently to specific inputs. Taking the results of our study as a starting point, the next stage is to identify the entirety of the targets of writers, readers, and erasers to comprehend the molecular effects of epigenetic modulation at the RNA molecule levels we observed during aging and according to the diet.

Considering the reversible nature of the epigenetic modifications, understanding all the above aspects will be necessary for the development of preventive and interventional approaches to improve well-being and healthy ageing.

## Data Availability

All data were reported in the manuscript and in the Supplementary Materials.

## Supplementary Materials

The following are available online at https://www.mdpi.com/article/10.3390/nu14061232/s1, Figure S1: Expression levels of writer (A), reader (B), and eraser (C) genes in the heart, liver, and kidney from rats of 27, 36, and 96 weeks of age fed with standard and low-calorie diet. Table S1: Description and functional annotation of the writer genes acquired from Rat Genome Database (RGD) according to the Gene Ontology. Table S2: Description and functional annotation of the reader genes acquired from Rat Genome Database (RGD) according to the Gene Ontology. Table S3: Description and functional annotation of the eraser genes acquired from Rat Genome Database (RGD) according to the Gene Ontology. Table S4. Nucleotide sequences (5′→3′) and annealing temperature of the primers used in the gene expression analysis. Table S5. Expression levels of writer, reader and eraser genes in the heart, liver, and kidney from rats of 27, 36, and 96 weeks of age, fed with standard and low-calorie diet. Table S6. Expression levels of writer, reader and eraser genes in the heart, liver, and kidney from rats of 27, 36, and 96 weeks of age, fed with standard and low-calorie diet.
