# Supplementary material for: Impact of Nutrition on Age-Related Epigenetic RNA Modifications in Rats
Source: Nutrients. 2022 Mar 15;14(6):1232. doi: 10.3390/nu14061232 (PMC8955587; doi:10.3390/nu14061232)
Supplement: Supplementary file 1 [file nutrients-14-01232-s001.zip › nutrients-1598442-supplementary.pdf]

Supplementary Materials

A

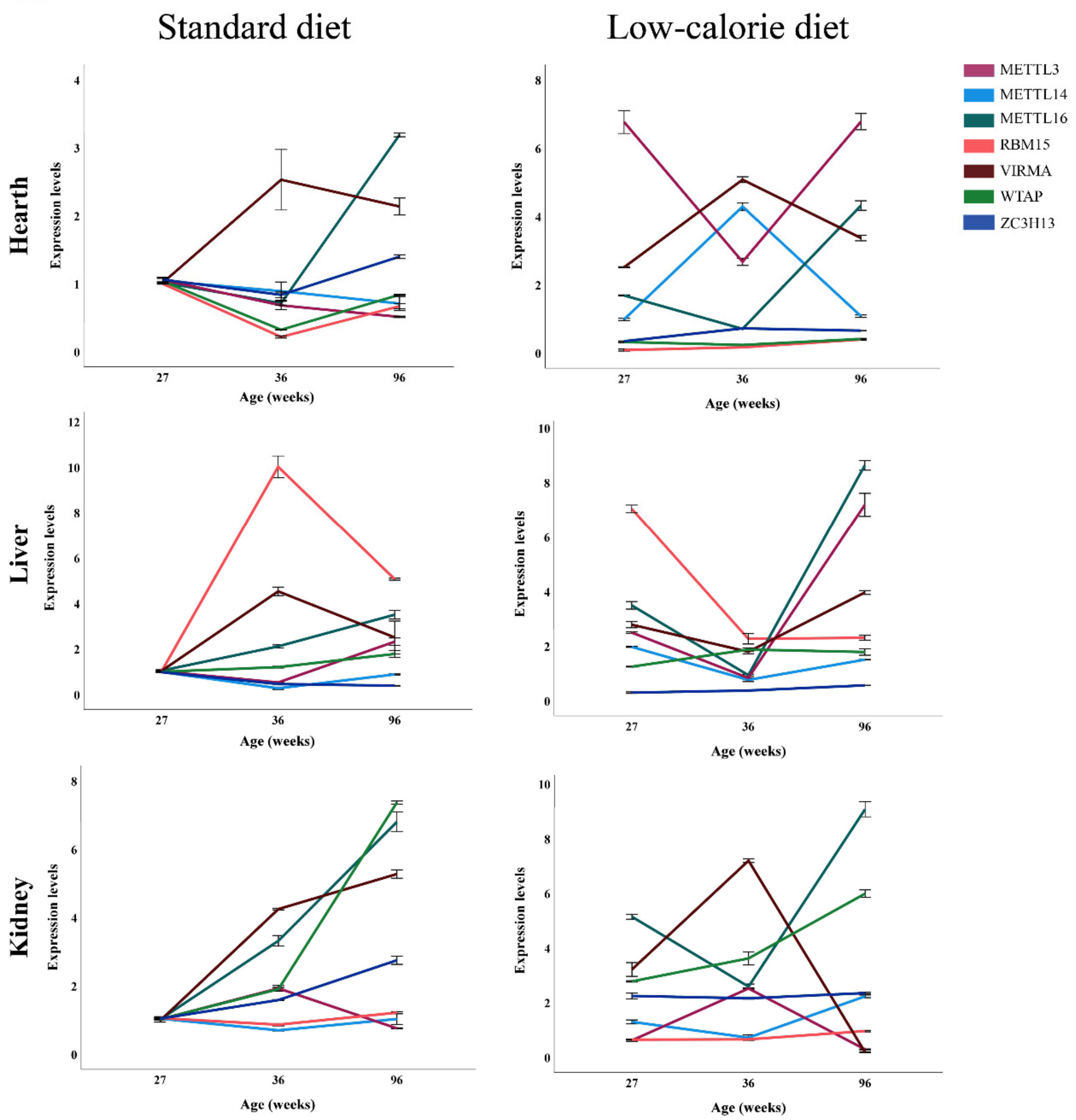

B

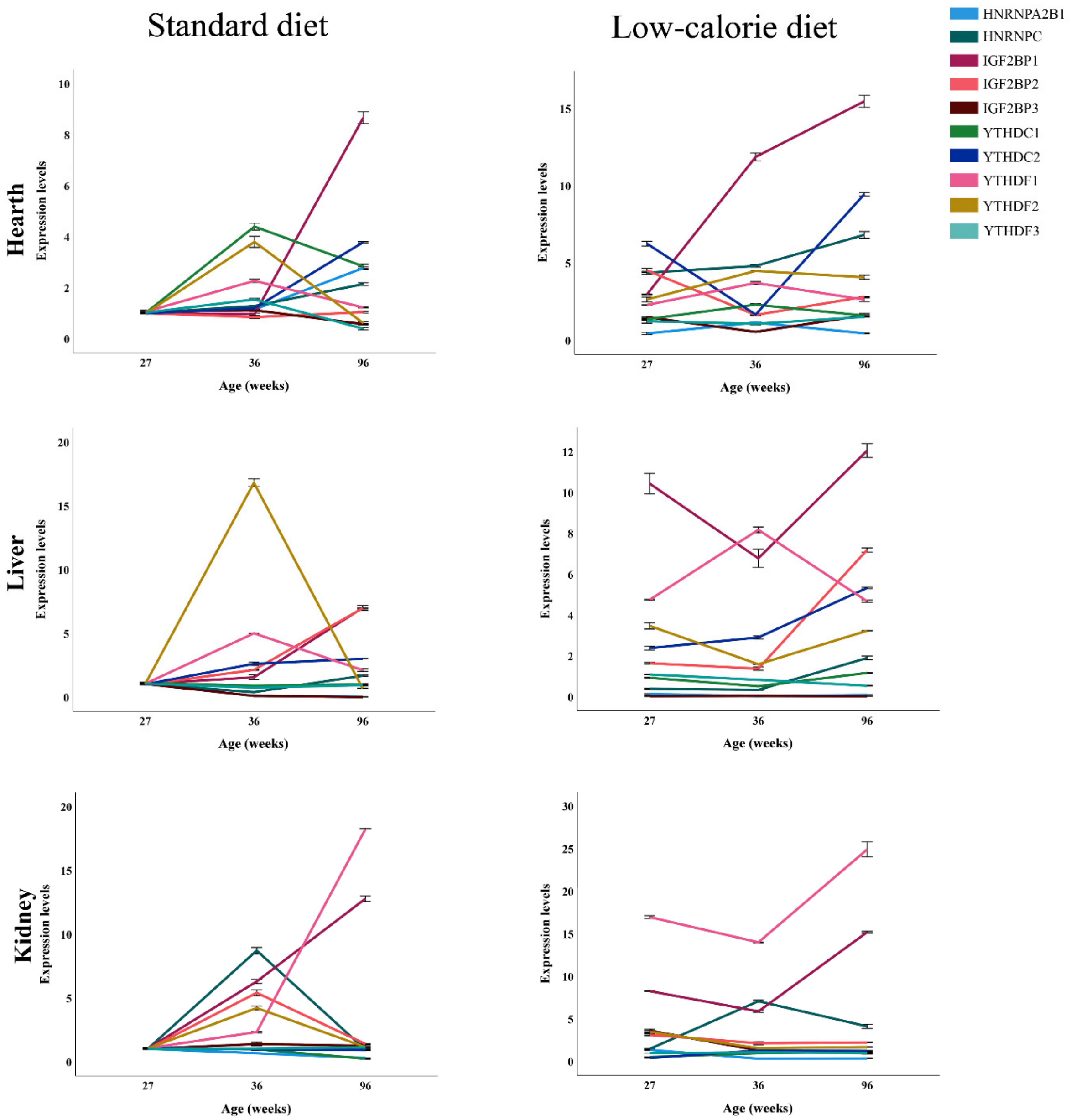
Standard diet
Low-calorie diet
HNRNPA2B1
HNRNPC
IGF2BP1
IGF2BP2
IGF2BP3
YTHDC1
YTHDC2
YTHDF1
YTHDF2
YTHDF3
Hearth
Liver
Kidney
Expression levels
Age (weeks)
27
36
96

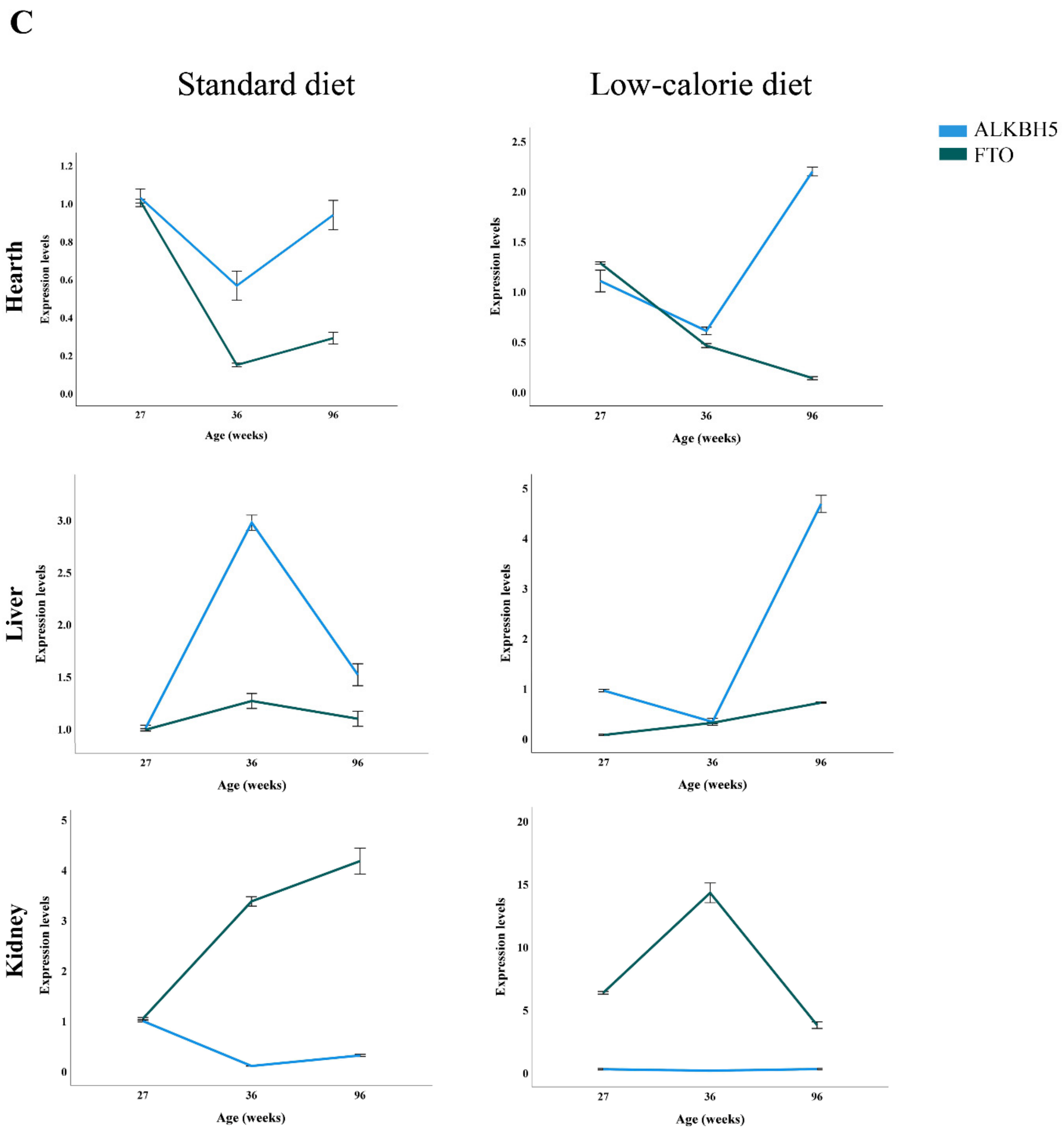

Figure S1. Expression levels of writer (**A**), reader (**B**), and eraser (**C**) genes in the heart, liver, and kidney from rats of 27, 36, and 96 weeks of age fed with standard and low-calorie diet. The values are reported as the mean of Relative Quantification values (RQ), measured in three independent triplicate experiments with standard deviation.

Table S1. Description and functional annotation of the Writers genes acquired from Rat Genome Database (RGD) according to the Gene Ontology. GO results are classified by the number of GO terms related to Biological Processes, Cellular Components, and Molecular Functions.

Function	Gene	Primer Forward sequence	Primer Reverse sequence	Annealing temperature (°C)
WRITERS	METTL3	CCAGCACAACGTCTATAGTCC	CGCTTTACCTCAATCAACTCCTG	60
	METTL14	TTGGGAGCTGAGAGTGCA	CCGTCTCTCCTTCATCCAGA	60
	METTL16	CAAGGACAAACCACCTGACTT	GTGGGAATTAGTCTCTCCAAAGG	59
	RBM15	CTGTGGGAGGCAATAAAGACA	CATCCGATGTTCACCAGTTTTG	59
	VIRMA	TGAAGATGAGGATGACTGCCA	TTCACGTTCCAAGTCAGCAA	59
	WTAP	TGGAGGGAAAGTACACAGACC	ACTCTTGCATCTCCTGTTCCT	59
	ZC3H13	GGAGGAAGGTCACAGTGGAA	GCTGCTGTAACCTTTCCCAC	60
READERS	HNRNPA2B1	ATTGATGGCAGGGTGGTTGA	TTTCCAGACTGCCTATCAGTA	61
	HNRNPC	ACTTGGACTATGACTTTCAACGC	CTGGCGTTTGGAAGGCAC	60
	IGF2BP1	TAGACGTGCACAGGAAGGAG	CGCCCTTCCTTTCCAATGAG	60
	IGF2BP2	CCTGGTGAAGACGGGCTAC	AGGCTTCAGATGTCCCTTCT	60
	IGF2BP3	GTTGGTGCCATCATCGGAAA	TCAACTTCCATCGGTTTCCCA	60
	YTHDC1	ACATGGGCTCCTGGTTTCC	GCAAGAGACACATTCTCATGGT	60
	YTHDC2	ACCGACTAAGTCAATCTCTTGGT	AGGCTCCTAACAGCATGTTTTG	60
	YTHDF1	TGACTTTGAGCCCTACCTTTCT	GTGAGATACGGGATGGGAGG	58
	YTHDF2	ACCTTACTTGAGCCCACAGG	TGGTAGAAAGTGGGGCTCTC	60
	YTHDF3	CTGACAGCTGCAGTGACAAA	TTCGGTTGAGGTTTTGCAGG	60
ERASER	ALKBH5	CATACGGCCTCAGGACATCA	TTCCAATCGTGGTGCATCTAA	58
	FTO	GGAGCTTGAAGACACTTGGC	TTGTGCAGTGTGAGAAAGGC	58

Table S2. Description and functional annotation of the Readers genes acquired from Rat Genome Database (RGD) according to the Gene Ontology. GO results are classified by the number of GO terms related to Biological Processes, Cellular Components, and Molecular Functions.

			27 weeks							36 weeks							96 weeks						
			Standard diet		Low-calorie diet					Standard diet		Low-calorie diet					Standard diet		Low-calorie diet				
		Gene	Mean	sd	Mean	sd	log2FC	p-value	Bonferroni-adjusted p-values	Mean	sd	Mean	sd	log2FC	p-value	Bonferroni-adjusted p-values	Mean	sd	Mean	sd	log2FC	p-value	Bonferroni-adjusted p-values
Heart	Writers	METTL3	1.056	0.048	6.764	0.583	2.680	0.003	0.057	0.682	0.110	2.669	0.183	1.970	0.000	0.006	0.512	0.018	6.774	0.413	3.720	0.001	0.019
		METTL14	1.041	0.068	0.984	0.058	-0.080	0.329	1.000	0.891	0.232	4.286	0.188	2.270	0.000	0.001	0.709	0.180	1.077	0.064	0.600	0.058	1.000
		METTL16	1.019	0.017	1.684	0.026	0.730	0.000	0.000	0.712	0.050	0.704	0.004	-0.020	0.805	1.000	3.187	0.048	4.318	0.241	0.440	0.012	0.228
		RBM15	0.999	0.002	0.085	0.048	-3.560	0.001	0.017	0.218	0.027	0.165	0.010	-0.400	0.067	1.000	0.669	0.066	0.393	0.030	-0.770	0.009	0.171
		VIRMA	1.015	0.013	2.511	0.025	1.310	0.000	0.000	2.529	0.771	5.074	0.143	1.000	0.026	0.494	2.135	0.215	3.366	0.140	0.660	0.002	0.038
		WTAP	1.036	0.074	0.320	0.033	-1.690	0.001	0.018	0.322	0.004	0.234	0.024	-0.460	0.020	0.380	0.837	0.015	0.410	0.028	-1.030	0.000	0.003
		ZC3H13	1.060	0.061	0.344	0.014	-1.630	0.002	0.038	0.836	0.072	0.719	0.025	-0.220	0.094	1.000	1.398	0.047	0.654	0.004	-1.100	0.001	0.019
	Readers	HNRNPA2B1	1.047	0.112	0.437	0.133	-1.260	0.004	0.076	1.145	0.115	1.143	0.040	0.000	0.981	1.000	2.768	0.087	0.446	0.052	-2.630	0.000	0.000
		HNRNPC	1.006	0.017	4.364	0.158	2.120	0.001	0.012	1.280	0.042	4.803	0.132	1.910	0.000	0.003	2.137	0.094	6.819	0.370	1.670	0.001	0.019
		IGF2BP1	0.999	0.030	2.962	0.045	1.570	0.000	0.000	0.957	0.084	11.858	0.440	3.630	0.000	0.007	8.658	0.404	15.456	0.669	0.840	0.000	0.007
		IGF2BP2	0.998	0.023	4.516	0.231	2.180	0.001	0.019	0.841	0.085	1.632	0.073	0.960	0.000	0.005	1.037	0.060	2.816	0.029	1.440	0.000	0.001
		IGF2BP3	1.050	0.045	1.493	0.069	0.510	0.001	0.019	1.114	0.066	0.542	0.056	-1.040	0.000	0.007	0.568	0.049	1.618	0.188	1.510	0.007	0.133
		YTHDC1	1.013	0.023	1.358	0.101	0.420	0.023	0.437	4.393	0.231	2.304	0.091	-0.930	0.001	0.019	2.834	0.146	1.592	0.058	-0.830	0.002	0.038
		YTHDC2	0.985	0.016	6.249	0.260	2.670	0.001	0.015	1.201	0.044	1.661	0.114	0.470	0.012	0.228	3.779	0.040	9.450	0.190	1.320	0.000	0.004
		YTHDF1	1.060	0.076	2.283	0.018	1.110	0.001	0.014	2.267	0.107	3.706	0.157	0.710	0.000	0.008	1.222	0.023	2.631	0.212	1.110	0.007	0.133
		YTHDF2	0.991	0.008	2.637	0.276	1.410	0.009	0.171	3.794	0.375	4.484	0.078	0.240	0.080	1.000	0.608	0.085	4.073	0.232	2.740	0.000	0.009
		YTHDF3	1.020	0.028	1.231	0.219	0.270	0.235	1.000	1.539	0.063	1.062	0.108	-0.540	0.006	0.114	0.391	0.083	1.506	0.034	1.950	0.000	0.009
	Erasers	ALKBH5	1.030	0.081	1.106	0.189	0.100	0.569	1.000	0.567	0.133	0.609	0.065	0.100	0.660	1.000	0.939	0.134	2.197	0.076	1.230	0.001	0.011
		FTO	1.012	0.018	1.284	0.020	0.340	0.000	0.001	0.149	0.017	0.462	0.034	1.630	0.001	0.017	0.291	0.053	0.138	0.027	-1.080	0.021	0.399
Liver	Writers	METTL3	1.007	0.015	2.492	0.048	1.310	0.000	0.002	0.524	0.024	0.832	0.106	0.670	0.032	0.608	2.431	0.259	7.181	0.730	1.560	0.004	0.076
		METTL14	0.988	0.023	1.969	0.028	0.990	0.000	0.000	0.270	0.087	0.761	0.108	1.500	0.004	0.076	0.896	0.040	1.502	0.011	0.750	0.001	0.013
		METTL16	1.045	0.067	3.488	0.238	1.740	0.002	0.038	2.109	0.123	0.935	0.057	-1.170	0.001	0.017	3.553	0.212	8.628	0.307	1.280	0.000	0.001
		RBM15	1.018	0.017	7.037	0.231	2.790	0.000	0.009	10.015	0.823	2.264	0.331	-2.150	0.001	0.019	4.804	0.442	2.303	0.154	-1.060	0.006	0.114
		VIRMA	1.035	0.040	2.781	0.195	1.430	0.003	0.057	4.524	0.325	1.791	0.122	-1.340	0.002	0.038	2.501	1.019	3.959	0.121	0.660	0.290	1.000
		WTAP	0.995	0.031	1.245	0.021	0.320	0.001	0.012	1.197	0.062	1.871	0.111	0.640	0.002	0.038	1.780	0.154	1.781	0.202	0.000	0.993	1.000

		**ZC3H13**	0.977	0.021	0.297	0.052	-1.720	0.001	0.010	0.457	0.019	0.372	0.011	-0.300	0.005	0.095	0.389	0.017	0.562	0.006	0.530	0.001	0.019
	**Readers**	**HNRNPA2B1**	1.032	0.079	0.130	0.018	-2.980	0.002	0.038	0.094	0.002	0.022	0.004	-2.110	0.000	0.001	0.025	0.010	0.080	0.023	1.710	0.035	0.665
		**HNRNPC**	1.012	0.019	0.381	0.047	-1.410	0.000	0.009	0.387	0.011	0.328	0.040	-0.240	0.114	1.000	1.673	0.076	1.893	0.159	0.180	0.125	1.000
		**IGF2BP1**	1.025	0.054	10.437	0.863	3.350	0.003	0.057	1.550	0.329	6.774	0.774	2.130	0.003	0.057	6.993	0.335	12.052	0.589	0.790	0.001	0.014
		**IGF2BP2**	0.977	0.021	1.636	0.067	0.740	0.002	0.038	2.128	0.058	1.368	0.139	-0.640	0.005	0.095	6.962	0.098	7.184	0.179	0.050	0.152	1.000
		**IGF2BP3**	1.028	0.030	0.021	0.032	-5.650	0.000	0.000	0.095	0.004	0.053	0.008	-0.840	0.004	0.076	0.004	0.003	0.009	0.003	1.280	0.108	1.000
		**YTHDC1**	1.104	0.096	0.921	0.053	-0.260	0.060	1.000	0.888	0.070	0.504	0.024	-0.820	0.006	0.114	0.995	0.076	1.159	0.024	0.220	0.054	1.000
		**YTHDC2**	1.000	0.000	2.375	0.162	1.250	0.162	1.000	2.611	0.236	2.898	0.141	0.150	0.160	1.000	3.008	0.040	5.321	0.076	0.820	0.000	0.000
		**YTHDF1**	1.039	0.035	4.740	0.057	2.190	0.000	0.000	5.000	0.011	8.172	0.234	0.710	0.002	0.038	2.114	0.176	4.673	0.093	1.140	0.000	0.004
		**YTHDF2**	1.016	0.031	3.466	0.269	1.770	0.004	0.076	16.799	0.508	1.585	0.029	-3.410	0.000	0.007	0.682	0.021	3.227	0.010	2.240	0.000	0.000
		**YTHDF3**	1.045	0.112	1.084	0.018	0.050	0.605	1.000	0.744	0.055	0.818	0.038	0.140	0.134	1.000	0.928	0.102	0.527	0.025	-0.810	0.017	0.323
	**Erasers**	**ALKBH5**	1.007	0.050	0.959	0.043	-0.070	0.269	1.000	2.974	0.126	0.335	0.119	-3.150	0.000	0.000	1.518	0.182	4.681	0.298	1.620	0.000	0.006
		**FTO**	0.992	0.017	0.075	0.023	-3.730	0.000	0.000	1.267	0.124	0.316	0.035	-2.000	0.003	0.057	1.097	0.125	0.719	0.017	-0.610	0.033	0.627
**Kidney**	**Writers**	**METTL3**	1.020	0.035	0.619	0.065	-0.720	0.002	0.038	1.926	0.150	2.517	0.031	0.390	0.017	0.323	0.756	0.029	0.288	0.029	-1.390	0.000	0.001
		**METTL14**	1.040	0.070	1.295	0.122	0.320	0.048	0.912	0.697	0.033	0.718	0.171	0.040	0.857	1.000	1.029	0.280	2.230	0.083	1.120	0.012	0.228
		**METTL16**	1.013	0.013	5.142	0.158	2.340	0.000	0.009	3.318	0.266	2.585	0.161	-0.360	0.022	0.418	6.798	0.500	9.075	0.479	0.420	0.005	0.095
		**RBM15**	1.054	0.075	0.643	0.026	-0.710	0.006	0.114	0.863	0.055	0.655	0.036	-0.400	0.008	0.152	1.215	0.055	0.958	0.047	-0.340	0.004	0.076
		**VIRMA**	0.978	0.070	3.212	0.436	1.720	0.011	0.209	4.247	0.043	7.193	0.121	0.760	0.000	0.003	5.272	0.214	0.182	0.016	-4.860	0.001	0.011
		**WTAP**	1.022	0.039	2.776	0.043	1.440	0.000	0.000	1.904	0.088	3.618	0.412	0.930	0.015	0.285	7.362	0.081	5.987	0.239	-0.300	0.005	0.095
		**ZC3H13**	1.039	0.064	2.243	0.184	1.110	0.004	0.076	1.584	0.022	2.158	0.054	0.450	0.001	0.018	2.748	0.208	2.349	0.043	-0.230	0.074	1.000
	**Readers**	**HNRNPA2B1**	1.004	0.024	1.338	0.082	0.410	0.014	0.266	0.656	0.059	0.306	0.007	-1.100	0.008	0.152	0.297	0.009	0.344	0.058	0.210	0.289	1.000
		**HNRNPC**	1.016	0.014	1.447	0.055	0.510	0.004	0.076	8.700	0.428	7.056	0.231	-0.300	0.009	0.171	0.929	0.111	4.097	0.417	2.140	0.004	0.076
		**IGF2BP1**	1.031	0.049	8.250	0.061	3.000	0.000	0.000	6.282	0.274	5.858	0.224	-0.100	0.110	1.000	12.768	0.377	15.167	0.170	0.250	0.003	0.057
		**IGF2BP2**	0.988	0.018	3.096	0.168	1.650	0.002	0.038	5.396	0.406	2.120	0.304	-1.350	0.001	0.010	1.382	0.028	2.210	0.043	0.680	0.000	0.001
		**IGF2BP3**	1.003	0.017	3.594	0.280	1.840	0.004	0.076	1.377	0.245	1.273	0.111	-0.110	0.553	1.000	1.242	0.127	0.929	0.148	-0.420	0.051	0.969
		**YTHDC1**	1.002	0.083	0.504	0.031	-0.990	0.004	0.076	0.969	0.065	0.949	0.028	-0.030	0.651	1.000	0.229	0.061	1.106	0.063	2.270	0.000	0.001
		**YTHDC2**	1.058	0.061	0.381	0.017	-1.470	0.001	0.019	0.959	0.145	1.264	0.035	0.400	0.061	1.000	0.907	0.039	1.211	0.104	0.420	0.025	0.475
		**YTHDF1**	1.050	0.046	16.944	0.284	4.010	0.000	0.001	2.320	0.092	13.976	0.053	2.590	0.000	0.000	18.228	0.085	24.891	1.518	0.450	0.017	0.323
		**YTHDF2**	1.040	0.066	3.466	0.237	1.740	0.002	0.038	4.203	0.255	1.530	0.036	-1.460	0.003	0.057	1.138	0.099	1.666	0.037	0.550	0.006	0.114
		**YTHDF3**	0.993	0.010	0.958	0.004	-0.050	0.015	0.285	1.015	0.088	1.038	0.084	0.030	0.761	1.000	1.115	0.047	0.980	0.040	-0.190	0.020	0.380

	**Erasers**	**ALKBH5**	0.999	0.032	0.283	0.104	-1.820	0.004	0.076	0.105	0.004	0.169	0.017	0.680	0.018	0.342	0.315	0.038	0.297	0.092	-0.090	0.770	1.000
		**FTO**	1.045	0.039	6.358	0.181	2.600	0.000	0.004	3.376	0.161	14.299	1.359	2.080	0.005	0.095	4.176	0.447	3.786	0.454	-0.140	0.349	1.000

Table S3. Description and functional annotation of the Erasers genes acquired from Rat Genome Database (RGD) according to the Gene Ontology. GO results are classified by the number of GO terms related to Biological Processes, Cellular Components, and Molecular Functions.

		Standard diet															
			36 vs 27					96 vs 27					96 vs 36				
			36	27				96	27				96	36			
		Gene	Mean	Mean	log2FC	p-value	Bonferroni-adjusted p-values	Mean	Mean	log2FC	p-value	Bonferroni-adjusted p-values	Mean	Mean	log2FC	p-value	Bonferroni-adjusted p-values
Heart	WRITERS	METTL3	0.682	1.056	-0.631	0.016	0.304	0.512	1.056	-1.044	0.001	0.018	0.512	0.682	-0.414	0.113	1.000
		METTL14	0.891	1.041	-0.224	0.379	1.000	0.709	1.041	-0.554	0.071	1.000	0.709	0.891	-0.330	0.347	1.000
		METTL16	0.712	1.019	-0.517	0.004	0.076	3.187	1.019	1.645	0.000	0.001	3.187	0.712	2.162	0.000	0.000
		RBM15	0.218	0.999	-2.196	0.000	0.007	0.669	0.999	-0.578	0.013	0.247	0.669	0.218	1.618	0.003	0.057
		VIRMA	2.529	1.015	1.317	0.077	1.000	2.135	1.015	1.073	0.012	0.228	2.135	2.529	-0.244	0.474	1.000
		WTAP	0.322	1.036	-1.686	0.004	0.076	0.837	1.036	-0.308	0.039	0.741	0.837	0.322	1.378	0.000	0.002
		ZC3H13	0.836	1.060	-0.342	0.015	0.285	1.398	1.060	0.399	0.002	0.038	1.398	0.836	0.742	0.001	0.015
	READERS	HNRNPA2B1	1.145	1.047	0.129	0.352	1.000	2.768	1.047	1.403	0.000	0.001	2.768	1.145	1.273	0.000	0.001
		HNRNPC	1.280	1.006	0.348	0.003	0.057	2.137	1.006	1.087	0.002	0.038	2.137	1.280	0.739	0.001	0.019
		IGF2BP1	0.957	0.998	-0.061	0.489	1.000	8.658	0.998	3.117	0.001	0.017	8.658	0.957	3.177	0.001	0.011
		IGF2BP2	0.841	0.998	-0.247	0.076	1.000	1.037	0.998	0.055	0.389	1.000	1.037	0.841	0.302	0.036	0.684
		IGF2BP3	1.114	1.050	0.085	0.249	1.000	0.568	1.050	-0.886	0.000	0.004	0.568	1.114	-0.972	0.001	0.010
		YTHDC1	4.393	1.013	2.117	0.001	0.019	2.834	1.013	1.484	0.002	0.038	2.834	4.393	-0.632	0.001	0.019
		YTHDC2	1.201	0.985	0.286	0.007	0.133	3.779	0.985	1.940	0.000	0.000	3.779	1.201	1.654	0.000	0.000
		YTHDF1	2.267	1.060	1.097	0.000	0.003	1.222	1.060	0.205	0.055	1.000	1.222	2.267	-0.892	0.002	0.038
		YTHDF2	3.794	0.991	1.937	0.006	0.114	0.608	0.991	-0.705	0.015	0.285	0.608	3.794	-2.642	0.003	0.057
		YTHDF3	1.539	1.020	0.593	0.001	0.019	0.391	1.020	-1.383	0.003	0.057	0.391	1.539	-1.977	0.000	0.001
	ERASERS	ALKBH5	0.567	1.030	-0.861	0.011	0.209	0.939	1.030	-0.133	0.382	1.000	0.939	0.567	0.728	0.027	0.513
		FTO	0.149	1.012	-2.764	0.000	0.000	0.291	1.012	-1.798	0.001	0.012	0.291	0.149	0.966	0.034	0.646
Liver	WRITERS	METTL3	0.524	1.007	-0.942	0.000	0.001	2.431	1.007	1.271	0.011	0.209	2.431	0.524	2.214	0.006	0.114
		METTL14	0.270	0.988	-1.872	0.003	0.057	0.896	0.988	-0.141	0.036	0.684	0.896	0.270	1.731	0.002	0.038
		METTL16	2.109	1.045	1.013	0.001	0.015	3.553	1.045	1.766	0.001	0.019	3.553	2.109	0.752	0.001	0.019
		RBM15	10.020	1.018	3.298	0.003	0.057	4.804	1.018	2.238	0.004	0.076	4.804	10.015	-1.060	0.002	0.038

		**VIRMA**	4.524	1.035	2.128	0.003	0.057	2.501	1.035	1.273	0.291	1.000	2.501	4.524	-0.855	0.200	1.000
		**WTAP**	1.197	0.995	0.267	0.016	0.304	1.780	0.995	0.839	0.010	0.190	1.780	1.197	0.572	0.013	0.247
		**ZC3H13**	0.457	0.976	-1.095	0.000	0.000	0.389	0.976	-1.327	0.000	0.000	0.389	0.457	-0.232	0.009	0.171
	**READERS**	**HNRNPA2B1**	0.094	1.032	-3.457	0.002	0.038	0.025	1.032	-5.367	0.002	0.038	0.025	0.094	-1.911	0.005	0.095
		**HNRNPC**	0.387	1.012	-1.387	0.000	0.000	1.673	1.012	0.725	0.003	0.057	1.673	0.387	2.112	0.001	0.018
		**IGF2BP1**	1.550	1.025	0.597	0.106	1.000	6.993	1.025	2.770	0.001	0.015	6.993	1.550	2.174	0.000	0.001
		**IGF2BP2**	2.128	0.976	1.125	0.000	0.004	6.962	0.976	2.835	0.000	0.001	6.962	2.128	1.710	0.000	0.000
		**IGF2BP3**	0.095	1.028	-3.436	0.000	0.005	0.004	1.028	-8.006	0.000	0.005	0.004	0.095	-4.570	0.000	0.000
		**YTHDC1**	0.888	1.104	-0.314	0.039	0.741	0.995	1.104	-0.150	0.204	1.000	0.995	0.888	0.164	0.146	1.000
		**YTHDC2**	2.611	1.000	1.385	0.007	0.133	3.008	1.000	1.589	0.000	0.000	3.008	2.611	0.204	0.096	1.000
		**YTHDF1**	5.000	1.039	2.267	0.000	0.000	2.114	1.039	1.025	0.007	0.133	2.114	5.000	-1.242	0.001	0.019
		**YTHDF2**	16.800	1.016	4.047	0.000	0.006	0.682	1.016	-0.575	0.000	0.005	0.682	16.799	-4.622	0.000	0.006
		**YTHDF3**	0.744	1.045	-0.490	0.027	0.513	0.928	1.045	-0.171	0.253	1.000	0.928	0.744	0.319	0.070	1.330
	**ERASERS**	**ALKBH5**	2.974	1.007	1.562	0.000	0.007	1.518	1.007	0.592	0.032	0.608	1.518	2.974	-0.970	0.001	0.012
		**FTO**	1.267	0.992	0.353	0.059	1.000	1.097	0.992	0.145	0.283	1.000	1.097	1.267	-0.208	0.170	1.000
**Kidney**	**WRITERS**	**METTL3**	1.926	1.020	0.917	0.007	0.133	0.756	1.020	-0.432	0.001	0.012	0.756	1.926	-1.349	0.004	0.076
		**METTL14**	0.697	1.040	-0.577	0.006	0.114	1.029	1.040	-0.015	0.953	1.000	1.029	0.697	0.562	0.175	1.000
		**METTL16**	3.318	1.013	1.712	0.004	0.076	6.798	1.013	2.746	0.002	0.038	6.798	3.318	1.035	0.002	0.038
		**RBM15**	0.863	1.054	-0.288	0.027	0.513	1.215	1.054	0.205	0.045	0.855	1.215	0.863	0.494	0.001	0.019
		**VIRMA**	4.247	0.978	2.119	0.000	0.000	5.272	0.978	2.430	0.000	0.005	5.272	4.247	0.312	0.012	0.228
		**WTAP**	1.904	1.022	0.898	0.001	0.016	7.362	1.022	2.849	0.000	0.000	7.362	1.904	1.951	0.000	0.000
		**ZC3H13**	1.584	1.039	0.608	0.002	0.038	2.748	1.039	1.403	0.003	0.057	2.748	1.584	0.795	0.010	0.190
	**READERS**	**HNRNPA2B1**	0.656	1.004	-0.614	0.004	0.076	0.297	1.004	-1.757	0.000	0.002	0.297	0.656	-1.143	0.008	0.152
		**HNRNPC**	8.700	1.016	3.098	0.001	0.019	0.929	1.016	-0.129	0.310	1.000	0.929	8.700	-3.227	0.001	0.010
		**IGF2BP1**	6.282	1.031	2.607	0.001	0.012	12.770	1.031	3.630	0.000	0.005	12.768	6.282	1.023	0.000	0.001
		**IGF2BP2**	5.396	0.988	2.449	0.003	0.057	1.382	0.988	0.484	0.000	0.002	1.382	5.396	-1.965	0.003	0.057
		**IGF2BP3**	1.377	1.003	0.457	0.118	1.000	1.242	1.003	0.308	0.080	1.000	1.242	1.377	-0.149	0.460	1.000
		**YTHDC1**	0.969	1.002	-0.048	0.618	1.000	0.229	1.002	-2.129	0.000	0.006	0.229	0.969	-2.081	0.000	0.003
		**YTHDC2**	0.959	1.058	-0.142	0.365	1.000	0.907	1.058	-0.222	0.029	0.551	0.907	0.959	-0.080	0.601	1.000
		**YTHDF1**	2.320	1.050	1.144	0.000	0.005	18.230	1.050	4.118	0.000	0.000	18.228	2.320	2.974	0.000	0.000

		**YTHDF2**	4.203	1.040	2.015	0.001	0.019	1.138	1.040	0.130	0.236	1.000	1.138	4.203	-1.885	0.001	0.014
		**YTHDF3**	1.015	0.993	0.032	0.711	1.000	1.115	0.993	0.167	0.041	0.779	1.115	1.015	0.136	0.178	1.000
	**ERASERS**	**ALKBH5**	0.105	0.999	-3.250	0.000	0.007	0.315	0.999	-1.665	0.000	0.000	0.315	0.105	1.585	0.010	0.190
		**FTO**	3.376	1.045	1.692	0.001	0.018	4.176	1.045	1.999	0.006	0.114	4.176	3.376	0.307	0.076	1.000

			**Low-calorie diet**														
			**36 vs 27**					**96 vs 27**					**96 vs 36**				
			**36**	**27**				**96**	**27**				**96**	**36**			
		**Gene**	**Mean**	**Mean**	**log2FC**	**p-value**	**Bonferroni-adjusted p-values**	**Mean**	**Mean**	**log2FC**	**p-value**	**Bonferroni-adjusted p-values**	**Mean**	**Mean**	**log2FC**	**p-value**	**Bonferroni-adjusted p-values**
**Heart**	**WRITERS**	**METTL3**	2.670	6.760	-1.342	0.004	0.076	6.774	6.764	0.002	0.982	1.000	6.774	2.669	1.344	0.001	0.017
		**METTL14**	4.290	0.980	2.123	0.000	0.008	1.077	0.984	0.130	0.133	1.000	1.077	4.286	-1.993	0.000	0.007
		**METTL16**	0.700	1.680	-1.258	0.000	0.003	4.318	1.684	1.358	0.003	0.057	4.318	0.704	2.617	0.001	0.019
		**RBM15**	0.170	0.090	0.957	0.094	1.000	0.393	0.085	2.209	0.002	0.038	0.393	0.165	1.252	0.003	0.057
		**VIRMA**	5.070	2.510	1.015	0.001	0.014	3.366	2.511	0.423	0.007	0.133	3.366	5.074	-0.592	0.000	0.002
		**WTAP**	0.230	0.320	-0.452	0.025	0.475	0.410	0.320	0.358	0.024	0.456	0.410	0.234	0.809	0.001	0.019
		**ZC3H13**	0.720	0.340	1.064	0.000	0.002	0.654	0.344	0.927	0.000	0.006	0.654	0.719	-0.137	0.043	0.817
	**READERS**	**HNRNPA2B1**	1.140	0.440	1.387	0.007	0.133	0.446	0.437	0.029	0.922	1.000	0.446	1.143	-1.358	0.000	0.001
		**HNRNPC**	4.800	4.360	0.138	0.022	0.418	6.819	4.364	0.644	0.003	0.057	6.819	4.803	0.506	0.006	0.114
		**IGF2BP1**	11.900	2.960	2.001	0.001	0.014	15.455	2.962	2.383	0.001	0.017	15.455	11.858	0.382	0.003	0.057
		**IGF2BP2**	1.630	4.520	-1.468	0.001	0.018	2.816	4.516	-0.681	0.006	0.114	2.816	1.632	0.787	0.000	0.006
		**IGF2BP3**	0.540	1.490	-1.462	0.000	0.001	1.618	1.493	0.116	0.372	1.000	1.618	0.542	1.578	0.006	0.114
		**YTHDC1**	2.300	1.360	0.764	0.000	0.005	1.592	1.357	0.230	0.036	0.684	1.592	2.304	-0.533	0.001	0.015
		**YTHDC2**	1.660	6.250	-1.912	0.000	0.004	9.450	6.249	0.597	0.000	0.002	9.450	1.661	2.508	0.000	0.000
		**YTHDF1**	3.710	2.280	0.699	0.004	0.076	2.631	2.283	0.205	0.104	1.000	2.631	3.706	-0.494	0.003	0.057
		**YTHDF2**	4.480	2.640	0.766	0.005	0.095	4.073	2.637	0.627	0.003	0.057	4.073	4.484	-0.139	0.079	1.000

		**YTHDF3**	1.060	1.230	-0.213	0.317	1.000	1.506	1.231	0.291	0.158	1.000	1.506	1.062	0.504	0.013	0.247
	**ERASERS**	**ALKBH5**	0.610	1.110	-0.861	0.034	0.646	2.197	1.106	0.990	0.004	0.076	2.197	0.609	1.851	0.000	0.000
		**FTO**	0.460	1.280	-1.475	0.000	0.000	0.138	1.284	-3.218	0.000	0.000	0.138	0.462	-1.743	0.000	0.005
**Liver**	**WRITERS**	**METTL3**	0.830	2.490	-1.583	0.000	0.005	7.181	2.492	1.527	0.008	0.152	7.181	0.832	3.110	0.004	0.076
		**METTL14**	0.760	1.970	-1.371	0.002	0.038	1.502	1.969	-0.391	0.000	0.006	1.502	0.761	0.981	0.007	0.133
		**METTL16**	0.940	3.490	-1.899	0.002	0.038	8.628	3.488	1.307	0.000	0.001	8.628	0.935	3.206	0.000	0.007
		**RBM15**	2.260	7.040	-1.636	0.000	0.001	2.303	7.037	-1.611	0.000	0.000	2.303	2.264	0.025	0.865	1.000
		**VIRMA**	1.790	2.780	-0.635	0.003	0.057	3.959	2.781	0.510	0.002	0.038	3.959	1.791	1.144	0.000	0.000
		**WTAP**	1.870	1.250	0.588	0.009	0.171	1.781	1.245	0.517	0.043	0.817	1.781	1.871	-0.071	0.546	1.000
	**READERS**	**ZC3H13**	0.370	0.300	0.325	0.123	1.000	0.562	0.297	0.920	0.012	0.228	0.562	0.372	0.595	0.000	0.002
		**HNRNPA2B1**	0.020	0.130	-2.563	0.007	0.133	0.080	0.130	-0.700	0.043	0.817	0.080	0.022	1.862	0.044	0.836
		**HNRNPC**	0.330	0.380	-0.216	0.207	1.000	1.893	0.381	2.313	0.002	0.038	1.893	0.328	2.529	0.002	0.038
		**IGF2BP1**	6.770	10.400	-0.624	0.006	0.114	12.052	10.437	0.208	0.063	1.000	12.052	6.774	0.831	0.001	0.019
		**IGF2BP2**	1.370	1.640	-0.259	0.060	1.140	7.184	1.636	2.135	0.000	0.001	7.184	1.367	2.394	0.000	0.000
		**IGF2BP3**	0.040	0.020	0.807	0.210	1.000	0.009	0.020	-1.152	0.588	1.000	0.009	0.035	-1.959	0.004	0.076
		**YTHDC1**	0.500	0.920	-0.870	0.002	0.038	1.159	0.921	0.332	0.007	0.133	1.159	0.504	1.201	0.000	0.000
		**YTHDC2**	2.900	2.380	0.287	0.014	0.266	5.321	2.375	1.164	0.000	0.003	5.321	2.898	0.877	0.000	0.002
		**YTHDF1**	8.170	4.740	0.786	0.001	0.017	4.673	4.740	-0.021	0.359	1.000	4.673	8.171	-0.806	0.000	0.007
		**YTHDF2**	1.590	3.470	-1.129	0.006	0.114	3.227	3.466	-0.103	0.262	1.000	3.227	1.585	1.026	0.000	0.000
		**YTHDF3**	0.820	1.080	-0.406	0.002	0.038	0.527	1.084	-1.040	0.000	0.000	0.527	0.818	-0.634	0.001	0.015
	**ERASERS**	**ALKBH5**	0.340	0.960	-1.517	0.007	0.133	4.681	0.959	2.287	0.002	0.038	4.681	0.335	3.805	0.000	0.008
		**FTO**	0.320	0.080	2.075	0.001	0.019	0.719	0.075	3.261	0.000	0.000	0.719	0.316	1.186	0.001	0.009
**Kidney**	**WRITERS**	**METTL3**	2.520	0.620	2.026	0.000	0.001	0.288	0.618	-1.102	0.005	0.095	0.288	2.517	-3.128	0.000	0.000
		**METTL14**	0.720	1.300	-0.851	0.011	0.209	2.230	1.295	0.784	0.001	0.014	2.230	0.718	1.635	0.001	0.019
		**METTL16**	2.590	5.140	-0.992	0.000	0.001	9.075	5.142	0.820	0.002	0.038	9.075	2.585	1.812	0.001	0.013
		**RBM15**	0.660	0.640	0.027	0.664	1.000	0.958	0.643	0.575	0.002	0.038	0.958	0.655	0.549	0.001	0.019
		**VIRMA**	7.190	3.210	1.163	0.002	0.038	0.182	3.212	-4.141	0.007	0.133	0.182	7.193	-5.305	0.000	0.001
		**WTAP**	3.620	2.780	0.382	0.070	1.330	5.987	2.776	1.109	0.001	0.019	5.987	3.618	0.727	0.003	0.057
		**ZC3H13**	2.160	2.240	-0.056	0.512	1.000	2.349	2.243	0.067	0.422	1.000	2.349	2.158	0.122	0.010	0.190
	**READERS**	**HNRNPA2B1**	7.060	1.450	2.286	0.002	0.038	4.097	1.447	1.502	0.000	0.003	4.097	7.056	-0.784	0.371	1.000

		**HNRNPC**	0.310	1.340	-2.128	0.000	0.006	0.344	1.338	-1.960	0.007	0.133	0.344	0.306	0.169	0.001	0.019
		**IGF2BP1**	5.860	8.250	-0.494	0.002	0.038	15.167	8.250	0.878	0.000	0.001	15.167	5.858	1.372	0.000	0.000
		**IGF2BP2**	2.120	3.100	-0.546	0.015	0.285	2.210	3.096	-0.486	0.008	0.152	2.210	2.120	0.060	0.663	1.000
		**IGF2BP3**	1.270	3.590	-1.497	0.002	0.038	0.929	3.594	-1.952	0.001	0.012	0.929	1.273	-0.454	0.036	0.684
		**YTHDC1**	0.950	0.500	0.913	0.000	0.001	1.106	0.504	1.134	0.001	0.015	1.106	0.949	0.221	0.034	0.646
		**YTHDC2**	1.260	0.380	1.730	0.000	0.001	1.211	0.381	1.668	0.004	0.076	1.211	1.264	-0.062	0.477	1.000
		**YTHDF1**	14.000	16.900	-0.278	0.002	0.038	24.891	16.944	0.555	0.010	0.190	24.891	13.976	0.833	0.006	0.114
		**YTHDF2**	1.530	3.470	-1.179	0.004	0.076	1.666	3.465	-1.056	0.005	0.095	1.666	1.530	0.123	0.010	0.190
		**YTHDF3**	1.040	0.960	0.116	0.241	1.000	0.980	0.958	0.033	0.454	1.000	0.980	1.038	-0.083	0.358	1.000
	**ERASERS**	**ALKBH5**	0.170	0.280	-0.744	0.195	1.000	0.296	0.283	0.065	0.879	1.000	0.296	0.169	0.809	0.135	1.000
		**FTO**	14.300	6.360	1.169	0.009	0.171	3.786	6.358	-0.748	0.005	0.095	3.786	14.299	-1.917	0.003	0.057

Table S4. Nucleotide sequences (5'→3') and annealing temperature of the primers used in the gene expression analysis.

Function	Gene	Primer Forward sequence	Primer Reverse sequence	Annealing temperature (°C)
**WRITERS**	*Mettl3*	CCAGCACAACGTCTATAGTCC	CGCTTTACCTCAATCAACTCCTG	60
	Mettl14	TTGGGAGCTGAGAGTGCA	CCGTCTCTCCTTCATCCAGA	60
	Mettl16	CAAGGACAAACCACCTGACTT	GTGGGAATTAGTCTCTCCAAAGG	59
	Rbm15	CTGTGGGAGGCAATAAAGACA	CATCCGATGTTCACCAGTTTTG	59
	Virma	TGAAGATGAGGATGACTGCCA	TTCACGTTCCAAGTCAGCAA	59
	Wtap	TGGAGGGAAAGTACACAGACC	ACTCTTGCATCTCCTGTTCCT	59
	Zc3h13	GGAGGAAGGTCACAGTGGAA	GCTGCTGTAACCTTTCCCAC	60
**READERS**	*Hnrnpa2b1*	ATTGATGGCAGGGTGGTTGA	TTTCCAGACTGCCTATCAGTA	61
	Hnrnpc	ACTTGGACTATGACTTTCAACGC	CTGGCGTTTGGAAGGCAC	60
	Igf2bp1	TAGACGTGCACAGGAAGGAG	CGCCCTTCCTTTCCAATGAG	60
	Igf2bp2	CCTGGTGAAGACGGGCTAC	AGGCTTCAGATGTCCCTTCT	60
	Igf2bp3	GTTGGTGCCATCATCGGAAA	TCAACTTCCATCGGTTTCCCA	60
	Ythdc1	ACATGGGCTCCTGGTTTCC	GCAAGAGACACATTCTCATGGT	60
	Ythdc2	ACCGACTAAGTCAATCTCTTGGT	AGGCTCCTAACAGCATGTTTTG	60
	Ythdf1	TGACTTTGAGCCCTACCTTTCT	GTGAGATACGGGATGGGAGG	58
	Ythdf2	ACCTTACTTGAGCCCACAGG	TGGTAGAAAGTGGGGCTCTC	60
	Ythdf3	CTGACAGCTGCAGTGACAAA	TTCGGTTGAGGTTTTGCAGG	60
**ERASERS**	*Alkbh5*	CATACGGCCTCAGGACATCA	TTCCAATCGTGGTGCATCTAA	58
	Fto	GGAGCTTGAAGACACTTGGC	TTGTGCAGTGTGAGAAAGGC	58
**HOUSEKEEPING GENE**	*Gapdh*	CCCCCAATGTATCCGTTGTG	TAGCCCAGGATGCCCTTTAGT	60

Table S5. Expression levels of writer, reader and eraser genes in the heart, liver, and kidney from rats of 27, 36, and 96 weeks of age, fed with standard and low-calorie diet. The values represent the mean of three independent triplicate experiments with standard deviation. log2FC (Fold Change) indicates the change in expression in the low-calorie diet compared with the standard diet.

			**27 weeks**							**36 weeks**							**96 weeks**						
			**Standard diet**		**Low-calorie diet**					**Standard diet**		**Low-calorie diet**					**Standard diet**		**Low-calorie diet**				
		**Gene**	**Mean**	**sd**	**Mean**	**sd**	**log2FC**	**p-value**	**Bonferroni-adjusted p-values**	**Mean**	**sd**	**Mean**	**sd**	**log2FC**	**p-value**	**Bonferroni-adjusted p-values**	**Mean**	**sd**	**Mean**	**sd**	**log2FC**	**p-value**	**Bonferroni-adjusted p-values**
**Heart**	**Writers**	**METTL3**	1.056	0.048	6.764	0.583	2.680	0.003	0.057	0.682	0.110	2.669	0.183	1.970	0.000	0.006	0.512	0.018	6.774	0.413	3.720	0.001	0.019
		**METTL14**	1.041	0.068	0.984	0.058	-0.080	0.329	1.000	0.891	0.232	4.286	0.188	2.270	0.000	0.001	0.709	0.180	1.077	0.064	0.600	0.058	1.000
		**METTL16**	1.019	0.017	1.684	0.026	0.730	0.000	0.000	0.712	0.050	0.704	0.004	-0.020	0.805	1.000	3.187	0.048	4.318	0.241	0.440	0.012	0.228
		**RBM15**	0.999	0.002	0.085	0.048	-3.560	0.001	0.017	0.218	0.027	0.165	0.010	-0.400	0.067	1.000	0.669	0.066	0.393	0.030	-0.770	0.009	0.171
		**VIRMA**	1.015	0.013	2.511	0.025	1.310	0.000	0.000	2.529	0.771	5.074	0.143	1.000	0.026	0.494	2.135	0.215	3.366	0.140	0.660	0.002	0.038
		**WTAP**	1.036	0.074	0.320	0.033	-1.690	0.001	0.018	0.322	0.004	0.234	0.024	-0.460	0.020	0.380	0.837	0.015	0.410	0.028	-1.030	0.000	0.003
		**ZC3H13**	1.060	0.061	0.344	0.014	-1.630	0.002	0.038	0.836	0.072	0.719	0.025	-0.220	0.094	1.000	1.398	0.047	0.654	0.004	-1.100	0.001	0.019
	**Readers**	**HNRNPA2B1**	1.047	0.112	0.437	0.133	-1.260	0.004	0.076	1.145	0.115	1.143	0.040	0.000	0.981	1.000	2.768	0.087	0.446	0.052	-2.630	0.000	0.000
		**HNRNPC**	1.006	0.017	4.364	0.158	2.120	0.001	0.012	1.280	0.042	4.803	0.132	1.910	0.000	0.003	2.137	0.094	6.819	0.370	1.670	0.001	0.019
		**IGF2BP1**	0.999	0.030	2.962	0.045	1.570	0.000	0.000	0.957	0.084	11.858	0.440	3.630	0.000	0.007	8.658	0.404	15.456	0.669	0.840	0.000	0.007
		**IGF2BP2**	0.998	0.023	4.516	0.231	2.180	0.001	0.019	0.841	0.085	1.632	0.073	0.960	0.000	0.005	1.037	0.060	2.816	0.029	1.440	0.000	0.001
		**IGF2BP3**	1.050	0.045	1.493	0.069	0.510	0.001	0.019	1.114	0.066	0.542	0.056	-1.040	0.000	0.007	0.568	0.049	1.618	0.188	1.510	0.007	0.133
		**YTHDC1**	1.013	0.023	1.358	0.101	0.420	0.023	0.437	4.393	0.231	2.304	0.091	-0.930	0.001	0.019	2.834	0.146	1.592	0.058	-0.830	0.002	0.038
		**YTHDC2**	0.985	0.016	6.249	0.260	2.670	0.001	0.015	1.201	0.044	1.661	0.114	0.470	0.012	0.228	3.779	0.040	9.450	0.190	1.320	0.000	0.004
		**YTHDF1**	1.060	0.076	2.283	0.018	1.110	0.001	0.014	2.267	0.107	3.706	0.157	0.710	0.000	0.008	1.222	0.023	2.631	0.212	1.110	0.007	0.133
		**YTHDF2**	0.991	0.008	2.637	0.276	1.410	0.009	0.171	3.794	0.375	4.484	0.078	0.240	0.080	1.000	0.608	0.085	4.073	0.232	2.740	0.000	0.009
		**YTHDF3**	1.020	0.028	1.231	0.219	0.270	0.235	1.000	1.539	0.063	1.062	0.108	-0.540	0.006	0.114	0.391	0.083	1.506	0.034	1.950	0.000	0.009
	**Erasers**	**ALKBH5**	1.030	0.081	1.106	0.189	0.100	0.569	1.000	0.567	0.133	0.609	0.065	0.100	0.660	1.000	0.939	0.134	2.197	0.076	1.230	0.001	0.011
		**FTO**	1.012	0.018	1.284	0.020	0.340	0.000	0.001	0.149	0.017	0.462	0.034	1.630	0.001	0.017	0.291	0.053	0.138	0.027	-1.080	0.021	0.399
**Liver**	**Writers**	**METTL3**	1.007	0.015	2.492	0.048	1.310	0.000	0.002	0.524	0.024	0.832	0.106	0.670	0.032	0.608	2.431	0.259	7.181	0.730	1.560	0.004	0.076
		**METTL14**	0.988	0.023	1.969	0.028	0.990	0.000	0.000	0.270	0.087	0.761	0.108	1.500	0.004	0.076	0.896	0.040	1.502	0.011	0.750	0.001	0.013
		**METTL16**	1.045	0.067	3.488	0.238	1.740	0.002	0.038	2.109	0.123	0.935	0.057	-1.170	0.001	0.017	3.553	0.212	8.628	0.307	1.280	0.000	0.001

		**RBM15**	1.018	0.017	7.037	0.231	2.790	0.000	0.009	10.015	0.823	2.264	0.331	-2.150	0.001	0.019	4.804	0.442	2.303	0.154	-1.060	0.006	0.114
		**VIRMA**	1.035	0.040	2.781	0.195	1.430	0.003	0.057	4.524	0.325	1.791	0.122	-1.340	0.002	0.038	2.501	1.019	3.959	0.121	0.660	0.290	1.000
		**WTAP**	0.995	0.031	1.245	0.021	0.320	0.001	0.012	1.197	0.062	1.871	0.111	0.640	0.002	0.038	1.780	0.154	1.781	0.202	0.000	0.993	1.000
		**ZC3H13**	0.977	0.021	0.297	0.052	-1.720	0.001	0.010	0.457	0.019	0.372	0.011	-0.300	0.005	0.095	0.389	0.017	0.562	0.006	0.530	0.001	0.019
	**Readers**	**HNRNPA2B1**	1.032	0.079	0.130	0.018	-2.980	0.002	0.038	0.094	0.002	0.022	0.004	-2.110	0.000	0.001	0.025	0.010	0.080	0.023	1.710	0.035	0.665
		**HNRNPC**	1.012	0.019	0.381	0.047	-1.410	0.000	0.009	0.387	0.011	0.328	0.040	-0.240	0.114	1.000	1.673	0.076	1.893	0.159	0.180	0.125	1.000
		**IGF2BP1**	1.025	0.054	10.437	0.863	3.350	0.003	0.057	1.550	0.329	6.774	0.774	2.130	0.003	0.057	6.993	0.335	12.052	0.589	0.790	0.001	0.014
		**IGF2BP2**	0.977	0.021	1.636	0.067	0.740	0.002	0.038	2.128	0.058	1.368	0.139	-0.640	0.005	0.095	6.962	0.098	7.184	0.179	0.050	0.152	1.000
		**IGF2BP3**	1.028	0.030	0.021	0.032	-5.650	0.000	0.000	0.095	0.004	0.053	0.008	-0.840	0.004	0.076	0.004	0.003	0.009	0.003	1.280	0.108	1.000
		**YTHDC1**	1.104	0.096	0.921	0.053	-0.260	0.060	1.000	0.888	0.070	0.504	0.024	-0.820	0.006	0.114	0.995	0.076	1.159	0.024	0.220	0.054	1.000
		**YTHDC2**	1.000	0.000	2.375	0.162	1.250	0.162	1.000	2.611	0.236	2.898	0.141	0.150	0.160	1.000	3.008	0.040	5.321	0.076	0.820	0.000	0.000
		**YTHDF1**	1.039	0.035	4.740	0.057	2.190	0.000	0.000	5.000	0.011	8.172	0.234	0.710	0.002	0.038	2.114	0.176	4.673	0.093	1.140	0.000	0.004
		**YTHDF2**	1.016	0.031	3.466	0.269	1.770	0.004	0.076	16.799	0.508	1.585	0.029	-3.410	0.000	0.007	0.682	0.021	3.227	0.010	2.240	0.000	0.000
		**YTHDF3**	1.045	0.112	1.084	0.018	0.050	0.605	1.000	0.744	0.055	0.818	0.038	0.140	0.134	1.000	0.928	0.102	0.527	0.025	-0.810	0.017	0.323
	**Erasers**	**ALKBH5**	1.007	0.050	0.959	0.043	-0.070	0.269	1.000	2.974	0.126	0.335	0.119	-3.150	0.000	0.000	1.518	0.182	4.681	0.298	1.620	0.000	0.006
		**FTO**	0.992	0.017	0.075	0.023	-3.730	0.000	0.000	1.267	0.124	0.316	0.035	-2.000	0.003	0.057	1.097	0.125	0.719	0.017	-0.610	0.033	0.627
**Kidney**	**Writers**	**METTL3**	1.020	0.035	0.619	0.065	-0.720	0.002	0.038	1.926	0.150	2.517	0.031	0.390	0.017	0.323	0.756	0.029	0.288	0.029	-1.390	0.000	0.001
		**METTL14**	1.040	0.070	1.295	0.122	0.320	0.048	0.912	0.697	0.033	0.718	0.171	0.040	0.857	1.000	1.029	0.280	2.230	0.083	1.120	0.012	0.228
		**METTL16**	1.013	0.013	5.142	0.158	2.340	0.000	0.009	3.318	0.266	2.585	0.161	-0.360	0.022	0.418	6.798	0.500	9.075	0.479	0.420	0.005	0.095
		**RBM15**	1.054	0.075	0.643	0.026	-0.710	0.006	0.114	0.863	0.055	0.655	0.036	-0.400	0.008	0.152	1.215	0.055	0.958	0.047	-0.340	0.004	0.076
		**VIRMA**	0.978	0.070	3.212	0.436	1.720	0.011	0.209	4.247	0.043	7.193	0.121	0.760	0.000	0.003	5.272	0.214	0.182	0.016	-4.860	0.001	0.011
		**WTAP**	1.022	0.039	2.776	0.043	1.440	0.000	0.000	1.904	0.088	3.618	0.412	0.930	0.015	0.285	7.362	0.081	5.987	0.239	-0.300	0.005	0.095
		**ZC3H13**	1.039	0.064	2.243	0.184	1.110	0.004	0.076	1.584	0.022	2.158	0.054	0.450	0.001	0.018	2.748	0.208	2.349	0.043	-0.230	0.074	1.000
	**Readers**	**HNRNPA2B1**	1.004	0.024	1.338	0.082	0.410	0.014	0.266	0.656	0.059	0.306	0.007	-1.100	0.008	0.152	0.297	0.009	0.344	0.058	0.210	0.289	1.000
		**HNRNPC**	1.016	0.014	1.447	0.055	0.510	0.004	0.076	8.700	0.428	7.056	0.231	-0.300	0.009	0.171	0.929	0.111	4.097	0.417	2.140	0.004	0.076
		**IGF2BP1**	1.031	0.049	8.250	0.061	3.000	0.000	0.000	6.282	0.274	5.858	0.224	-0.100	0.110	1.000	12.768	0.377	15.167	0.170	0.250	0.003	0.057
		**IGF2BP2**	0.988	0.018	3.096	0.168	1.650	0.002	0.038	5.396	0.406	2.120	0.304	-1.350	0.001	0.010	1.382	0.028	2.210	0.043	0.680	0.000	0.001
		**IGF2BP3**	1.003	0.017	3.594	0.280	1.840	0.004	0.076	1.377	0.245	1.273	0.111	-0.110	0.553	1.000	1.242	0.127	0.929	0.148	-0.420	0.051	0.969
		**YTHDC1**	1.002	0.083	0.504	0.031	-0.990	0.004	0.076	0.969	0.065	0.949	0.028	-0.030	0.651	1.000	0.229	0.061	1.106	0.063	2.270	0.000	0.001
		**YTHDC2**	1.058	0.061	0.381	0.017	-1.470	0.001	0.019	0.959	0.145	1.264	0.035	0.400	0.061	1.000	0.907	0.039	1.211	0.104	0.420	0.025	0.475

		**YTHDF1**	1.050	0.046	16.944	0.284	4.010	0.000	0.001	2.320	0.092	13.976	0.053	2.590	0.000	0.000	18.228	0.085	24.891	1.518	0.450	0.017	0.323
		**YTHDF2**	1.040	0.066	3.466	0.237	1.740	0.002	0.038	4.203	0.255	1.530	0.036	-1.460	0.003	0.057	1.138	0.099	1.666	0.037	0.550	0.006	0.114
		**YTHDF3**	0.993	0.010	0.958	0.004	-0.050	0.015	0.285	1.015	0.088	1.038	0.084	0.030	0.761	1.000	1.115	0.047	0.980	0.040	-0.190	0.020	0.380
	**Erasers**	**ALKBH5**	0.999	0.032	0.283	0.104	-1.820	0.004	0.076	0.105	0.004	0.169	0.017	0.680	0.018	0.342	0.315	0.038	0.297	0.092	-0.090	0.770	1.000
		**FTO**	1.045	0.039	6.358	0.181	2.600	0.000	0.004	3.376	0.161	14.299	1.359	2.080	0.005	0.095	4.176	0.447	3.786	0.454	-0.140	0.349	1.000

Table S6. Expression levels of writer, reader and eraser genes in the heart, liver and kidney from rats of 27, 36, and 96 weeks of age, fed with standard and low-calorie diet. The values represent the mean of three independent triplicate experiments with standard deviation. log2FC (Fold Change) indicates the change in expression from 36 to 27, 96 to 27 and 96 to 36 weeks of age.

			Standard diet														
			36 vs 27					96 vs 27					96 vs 36				
			36	27				96	27				96	36			
		Gene	Mean	Mean	log2FC	p-value	Bonferroni-adjusted p-values	Mean	Mean	log2FC	p-value	Bonferroni-adjusted p-values	Mean	Mean	log2FC	p-value	Bonferroni-adjusted p-values
Heart	WRITERS	METTL3	0.682	1.056	-0.631	0.016	0.304	0.512	1.056	-1.044	0.001	0.018	0.512	0.682	-0.414	0.113	1.000
		METTL14	0.891	1.041	-0.224	0.379	1.000	0.709	1.041	-0.554	0.071	1.000	0.709	0.891	-0.330	0.347	1.000
		METTL16	0.712	1.019	-0.517	0.004	0.076	3.187	1.019	1.645	0.000	0.001	3.187	0.712	2.162	0.000	0.000
		RBM15	0.218	0.999	-2.196	0.000	0.007	0.669	0.999	-0.578	0.013	0.247	0.669	0.218	1.618	0.003	0.057
		VIRMA	2.529	1.015	1.317	0.077	1.000	2.135	1.015	1.073	0.012	0.228	2.135	2.529	-0.244	0.474	1.000
		WTAP	0.322	1.036	-1.686	0.004	0.076	0.837	1.036	-0.308	0.039	0.741	0.837	0.322	1.378	0.000	0.002
		ZC3H13	0.836	1.060	-0.342	0.015	0.285	1.398	1.060	0.399	0.002	0.038	1.398	0.836	0.742	0.001	0.015
	READERS	HNRNPA2B1	1.145	1.047	0.129	0.352	1.000	2.768	1.047	1.403	0.000	0.001	2.768	1.145	1.273	0.000	0.001
		HNRNPC	1.280	1.006	0.348	0.003	0.057	2.137	1.006	1.087	0.002	0.038	2.137	1.280	0.739	0.001	0.019
		IGF2BP1	0.957	0.998	-0.061	0.489	1.000	8.658	0.998	3.117	0.001	0.017	8.658	0.957	3.177	0.001	0.011
		IGF2BP2	0.841	0.998	-0.247	0.076	1.000	1.037	0.998	0.055	0.389	1.000	1.037	0.841	0.302	0.036	0.684
		IGF2BP3	1.114	1.050	0.085	0.249	1.000	0.568	1.050	-0.886	0.000	0.004	0.568	1.114	-0.972	0.001	0.010
		YTHDC1	4.393	1.013	2.117	0.001	0.019	2.834	1.013	1.484	0.002	0.038	2.834	4.393	-0.632	0.001	0.019
		YTHDC2	1.201	0.985	0.286	0.007	0.133	3.779	0.985	1.940	0.000	0.000	3.779	1.201	1.654	0.000	0.000
		YTHDF1	2.267	1.060	1.097	0.000	0.003	1.222	1.060	0.205	0.055	1.000	1.222	2.267	-0.892	0.002	0.038
		YTHDF2	3.794	0.991	1.937	0.006	0.114	0.608	0.991	-0.705	0.015	0.285	0.608	3.794	-2.642	0.003	0.057
		YTHDF3	1.539	1.020	0.593	0.001	0.019	0.391	1.020	-1.383	0.003	0.057	0.391	1.539	-1.977	0.000	0.001
	ERASERS	ALKBH5	0.567	1.030	-0.861	0.011	0.209	0.939	1.030	-0.133	0.382	1.000	0.939	0.567	0.728	0.027	0.513
		FTO	0.149	1.012	-2.764	0.000	0.000	0.291	1.012	-1.798	0.001	0.012	0.291	0.149	0.966	0.034	0.646
Liver	WRITERS	METTL3	0.524	1.007	-0.942	0.000	0.001	2.431	1.007	1.271	0.011	0.209	2.431	0.524	2.214	0.006	0.114

		**METTL14**	0.270	0.988	-1.872	0.003	0.057	0.896	0.988	-0.141	0.036	0.684	0.896	0.270	1.731	0.002	0.038
		**METTL16**	2.109	1.045	1.013	0.001	0.015	3.553	1.045	1.766	0.001	0.019	3.553	2.109	0.752	0.001	0.019
		**RBM15**	10.020	1.018	3.298	0.003	0.057	4.804	1.018	2.238	0.004	0.076	4.804	10.015	-1.060	0.002	0.038
		**VIRMA**	4.524	1.035	2.128	0.003	0.057	2.501	1.035	1.273	0.291	1.000	2.501	4.524	-0.855	0.200	1.000
		**WTAP**	1.197	0.995	0.267	0.016	0.304	1.780	0.995	0.839	0.010	0.190	1.780	1.197	0.572	0.013	0.247
		**ZC3H13**	0.457	0.976	-1.095	0.000	0.000	0.389	0.976	-1.327	0.000	0.000	0.389	0.457	-0.232	0.009	0.171
	**READERS**	**HNRNPA2B1**	0.094	1.032	-3.457	0.002	0.038	0.025	1.032	-5.367	0.002	0.038	0.025	0.094	-1.911	0.005	0.095
		**HNRNPC**	0.387	1.012	-1.387	0.000	0.000	1.673	1.012	0.725	0.003	0.057	1.673	0.387	2.112	0.001	0.018
		**IGF2BP1**	1.550	1.025	0.597	0.106	1.000	6.993	1.025	2.770	0.001	0.015	6.993	1.550	2.174	0.000	0.001
		**IGF2BP2**	2.128	0.976	1.125	0.000	0.004	6.962	0.976	2.835	0.000	0.001	6.962	2.128	1.710	0.000	0.000
		**IGF2BP3**	0.095	1.028	-3.436	0.000	0.005	0.004	1.028	-8.006	0.000	0.005	0.004	0.095	-4.570	0.000	0.000
		**YTHDC1**	0.888	1.104	-0.314	0.039	0.741	0.995	1.104	-0.150	0.204	1.000	0.995	0.888	0.164	0.146	1.000
		**YTHDC2**	2.611	1.000	1.385	0.007	0.133	3.008	1.000	1.589	0.000	0.000	3.008	2.611	0.204	0.096	1.000
		**YTHDF1**	5.000	1.039	2.267	0.000	0.000	2.114	1.039	1.025	0.007	0.133	2.114	5.000	-1.242	0.001	0.019
		**YTHDF2**	16.800	1.016	4.047	0.000	0.006	0.682	1.016	-0.575	0.000	0.005	0.682	16.799	-4.622	0.000	0.006
		**YTHDF3**	0.744	1.045	-0.490	0.027	0.513	0.928	1.045	-0.171	0.253	1.000	0.928	0.744	0.319	0.070	1.330
	**ERASERS**	**ALKBH5**	2.974	1.007	1.562	0.000	0.007	1.518	1.007	0.592	0.032	0.608	1.518	2.974	-0.970	0.001	0.012
		**FTO**	1.267	0.992	0.353	0.059	1.000	1.097	0.992	0.145	0.283	1.000	1.097	1.267	-0.208	0.170	1.000
**Kidney**	**WRITERS**	**METTL3**	1.926	1.020	0.917	0.007	0.133	0.756	1.020	-0.432	0.001	0.012	0.756	1.926	-1.349	0.004	0.076
		**METTL14**	0.697	1.040	-0.577	0.006	0.114	1.029	1.040	-0.015	0.953	1.000	1.029	0.697	0.562	0.175	1.000
		**METTL16**	3.318	1.013	1.712	0.004	0.076	6.798	1.013	2.746	0.002	0.038	6.798	3.318	1.035	0.002	0.038
		**RBM15**	0.863	1.054	-0.288	0.027	0.513	1.215	1.054	0.205	0.045	0.855	1.215	0.863	0.494	0.001	0.019
		**VIRMA**	4.247	0.978	2.119	0.000	0.000	5.272	0.978	2.430	0.000	0.005	5.272	4.247	0.312	0.012	0.228
		**WTAP**	1.904	1.022	0.898	0.001	0.016	7.362	1.022	2.849	0.000	0.000	7.362	1.904	1.951	0.000	0.000
		**ZC3H13**	1.584	1.039	0.608	0.002	0.038	2.748	1.039	1.403	0.003	0.057	2.748	1.584	0.795	0.010	0.190
	**READERS**	**HNRNPA2B1**	0.656	1.004	-0.614	0.004	0.076	0.297	1.004	-1.757	0.000	0.002	0.297	0.656	-1.143	0.008	0.152
		**HNRNPC**	8.700	1.016	3.098	0.001	0.019	0.929	1.016	-0.129	0.310	1.000	0.929	8.700	-3.227	0.001	0.010
		**IGF2BP1**	6.282	1.031	2.607	0.001	0.012	12.770	1.031	3.630	0.000	0.005	12.768	6.282	1.023	0.000	0.001
		**IGF2BP2**	5.396	0.988	2.449	0.003	0.057	1.382	0.988	0.484	0.000	0.002	1.382	5.396	-1.965	0.003	0.057
		**IGF2BP3**	1.377	1.003	0.457	0.118	1.000	1.242	1.003	0.308	0.080	1.000	1.242	1.377	-0.149	0.460	1.000

		**YTHDC1**	0.969	1.002	-0.048	0.618	1.000	0.229	1.002	-2.129	0.000	0.006	0.229	0.969	-2.081	0.000	0.003
		**YTHDC2**	0.959	1.058	-0.142	0.365	1.000	0.907	1.058	-0.222	0.029	0.551	0.907	0.959	-0.080	0.601	1.000
		**YTHDF1**	2.320	1.050	1.144	0.000	0.005	18.230	1.050	4.118	0.000	0.000	18.228	2.320	2.974	0.000	0.000
		**YTHDF2**	4.203	1.040	2.015	0.001	0.019	1.138	1.040	0.130	0.236	1.000	1.138	4.203	-1.885	0.001	0.014
		**YTHDF3**	1.015	0.993	0.032	0.711	1.000	1.115	0.993	0.167	0.041	0.779	1.115	1.015	0.136	0.178	1.000
	**ERASERS**	**ALKBH5**	0.105	0.999	-3.250	0.000	0.007	0.315	0.999	-1.665	0.000	0.000	0.315	0.105	1.585	0.010	0.190
		**FTO**	3.376	1.045	1.692	0.001	0.018	4.176	1.045	1.999	0.006	0.114	4.176	3.376	0.307	0.076	1.000

			**Low-calorie diet**														
			**36 vs 27**					**96 vs 27**					**96 vs 36**				
			**36**	**27**				**96**	**27**				**96**	**36**			
		**Gene**	**Mean**	**Mean**	**log2FC**	**p-value**	**Bonferroni-adjusted p-values**	**Mean**	**Mean**	**log2FC**	**p-value**	**Bonferroni-adjusted p-values**	**Mean**	**Mean**	**log2FC**	**p-value**	**Bonferroni-adjusted p-values**
**Heart**	**WRITERS**	**METTL3**	2.670	6.760	-1.342	0.004	0.076	6.774	6.764	0.002	0.982	1.000	6.774	2.669	1.344	0.001	0.017
		**METTL14**	4.290	0.980	2.123	0.000	0.008	1.077	0.984	0.130	0.133	1.000	1.077	4.286	-1.993	0.000	0.007
		**METTL16**	0.700	1.680	-1.258	0.000	0.003	4.318	1.684	1.358	0.003	0.057	4.318	0.704	2.617	0.001	0.019
		**RBM15**	0.170	0.090	0.957	0.094	1.000	0.393	0.085	2.209	0.002	0.038	0.393	0.165	1.252	0.003	0.057
		**VIRMA**	5.070	2.510	1.015	0.001	0.014	3.366	2.511	0.423	0.007	0.133	3.366	5.074	-0.592	0.000	0.002
		**WTAP**	0.230	0.320	-0.452	0.025	0.475	0.410	0.320	0.358	0.024	0.456	0.410	0.234	0.809	0.001	0.019
		**ZC3H13**	0.720	0.340	1.064	0.000	0.002	0.654	0.344	0.927	0.000	0.006	0.654	0.719	-0.137	0.043	0.817
	**READERS**	**HNRNPA2B1**	1.140	0.440	1.387	0.007	0.133	0.446	0.437	0.029	0.922	1.000	0.446	1.143	-1.358	0.000	0.001
		**HNRNPC**	4.800	4.360	0.138	0.022	0.418	6.819	4.364	0.644	0.003	0.057	6.819	4.803	0.506	0.006	0.114
		**IGF2BP1**	11.900	2.960	2.001	0.001	0.014	15.455	2.962	2.383	0.001	0.017	15.455	11.858	0.382	0.003	0.057
		**IGF2BP2**	1.630	4.520	-1.468	0.001	0.018	2.816	4.516	-0.681	0.006	0.114	2.816	1.632	0.787	0.000	0.006
		**IGF2BP3**	0.540	1.490	-1.462	0.000	0.001	1.618	1.493	0.116	0.372	1.000	1.618	0.542	1.578	0.006	0.114
		**YTHDC1**	2.300	1.360	0.764	0.000	0.005	1.592	1.357	0.230	0.036	0.684	1.592	2.304	-0.533	0.001	0.015

		**YTHDC2**	1.660	6.250	-1.912	0.000	0.004	9.450	6.249	0.597	0.000	0.002	9.450	1.661	2.508	0.000	0.000
		**YTHDF1**	3.710	2.280	0.699	0.004	0.076	2.631	2.283	0.205	0.104	1.000	2.631	3.706	-0.494	0.003	0.057
		**YTHDF2**	4.480	2.640	0.766	0.005	0.095	4.073	2.637	0.627	0.003	0.057	4.073	4.484	-0.139	0.079	1.000
		**YTHDF3**	1.060	1.230	-0.213	0.317	1.000	1.506	1.231	0.291	0.158	1.000	1.506	1.062	0.504	0.013	0.247
	**ERASERS**	**ALKBH5**	0.610	1.110	-0.861	0.034	0.646	2.197	1.106	0.990	0.004	0.076	2.197	0.609	1.851	0.000	0.000
		**FTO**	0.460	1.280	-1.475	0.000	0.000	0.138	1.284	-3.218	0.000	0.000	0.138	0.462	-1.743	0.000	0.005
**Liver**	**WRITERS**	**METTL3**	0.830	2.490	-1.583	0.000	0.005	7.181	2.492	1.527	0.008	0.152	7.181	0.832	3.110	0.004	0.076
		**METTL14**	0.760	1.970	-1.371	0.002	0.038	1.502	1.969	-0.391	0.000	0.006	1.502	0.761	0.981	0.007	0.133
		**METTL16**	0.940	3.490	-1.899	0.002	0.038	8.628	3.488	1.307	0.000	0.001	8.628	0.935	3.206	0.000	0.007
		**RBM15**	2.260	7.040	-1.636	0.000	0.001	2.303	7.037	-1.611	0.000	0.000	2.303	2.264	0.025	0.865	1.000
		**VIRMA**	1.790	2.780	-0.635	0.003	0.057	3.959	2.781	0.510	0.002	0.038	3.959	1.791	1.144	0.000	0.000
		**WTAP**	1.870	1.250	0.588	0.009	0.171	1.781	1.245	0.517	0.043	0.817	1.781	1.871	-0.071	0.546	1.000
	**READERS**	**ZC3H13**	0.370	0.300	0.325	0.123	1.000	0.562	0.297	0.920	0.012	0.228	0.562	0.372	0.595	0.000	0.002
		**HNRNPA2B1**	0.020	0.130	-2.563	0.007	0.133	0.080	0.130	-0.700	0.043	0.817	0.080	0.022	1.862	0.044	0.836
		**HNRNPC**	0.330	0.380	-0.216	0.207	1.000	1.893	0.381	2.313	0.002	0.038	1.893	0.328	2.529	0.002	0.038
		**IGF2BP1**	6.770	10.400	-0.624	0.006	0.114	12.052	10.437	0.208	0.063	1.000	12.052	6.774	0.831	0.001	0.019
		**IGF2BP2**	1.370	1.640	-0.259	0.060	1.140	7.184	1.636	2.135	0.000	0.001	7.184	1.367	2.394	0.000	0.000
		**IGF2BP3**	0.040	0.020	0.807	0.210	1.000	0.009	0.020	-1.152	0.588	1.000	0.009	0.035	-1.959	0.004	0.076
		**YTHDC1**	0.500	0.920	-0.870	0.002	0.038	1.159	0.921	0.332	0.007	0.133	1.159	0.504	1.201	0.000	0.000
		**YTHDC2**	2.900	2.380	0.287	0.014	0.266	5.321	2.375	1.164	0.000	0.003	5.321	2.898	0.877	0.000	0.002
		**YTHDF1**	8.170	4.740	0.786	0.001	0.017	4.673	4.740	-0.021	0.359	1.000	4.673	8.171	-0.806	0.000	0.007
		**YTHDF2**	1.590	3.470	-1.129	0.006	0.114	3.227	3.466	-0.103	0.262	1.000	3.227	1.585	1.026	0.000	0.000
		**YTHDF3**	0.820	1.080	-0.406	0.002	0.038	0.527	1.084	-1.040	0.000	0.000	0.527	0.818	-0.634	0.001	0.015
	**ERASERS**	**ALKBH5**	0.340	0.960	-1.517	0.007	0.133	4.681	0.959	2.287	0.002	0.038	4.681	0.335	3.805	0.000	0.008
		**FTO**	0.320	0.080	2.075	0.001	0.019	0.719	0.075	3.261	0.000	0.000	0.719	0.316	1.186	0.001	0.009
**Kidney**	**WRITERS**	**METTL3**	2.520	0.620	2.026	0.000	0.001	0.288	0.618	-1.102	0.005	0.095	0.288	2.517	-3.128	0.000	0.000
		**METTL14**	0.720	1.300	-0.851	0.011	0.209	2.230	1.295	0.784	0.001	0.014	2.230	0.718	1.635	0.001	0.019
		**METTL16**	2.590	5.140	-0.992	0.000	0.001	9.075	5.142	0.820	0.002	0.038	9.075	2.585	1.812	0.001	0.013
		**RBM15**	0.660	0.640	0.027	0.664	1.000	0.958	0.643	0.575	0.002	0.038	0.958	0.655	0.549	0.001	0.019
		**VIRMA**	7.190	3.210	1.163	0.002	0.038	0.182	3.212	-4.141	0.007	0.133	0.182	7.193	-5.305	0.000	0.001

	**WTAP**	3.620	2.780	0.382	0.070	1.330	5.987	2.776	1.109	0.001	0.019	5.987	3.618	0.727	0.003	0.057
	**ZC3H13**	2.160	2.240	-0.056	0.512	1.000	2.349	2.243	0.067	0.422	1.000	2.349	2.158	0.122	0.010	0.190
**READERS**	**HNRNPA2B1**	7.060	1.450	2.286	0.002	0.038	4.097	1.447	1.502	0.000	0.003	4.097	7.056	-0.784	0.371	1.000
	**HNRNPC**	0.310	1.340	-2.128	0.000	0.006	0.344	1.338	-1.960	0.007	0.133	0.344	0.306	0.169	0.001	0.019
	**IGF2BP1**	5.860	8.250	-0.494	0.002	0.038	15.167	8.250	0.878	0.000	0.001	15.167	5.858	1.372	0.000	0.000
	**IGF2BP2**	2.120	3.100	-0.546	0.015	0.285	2.210	3.096	-0.486	0.008	0.152	2.210	2.120	0.060	0.663	1.000
	**IGF2BP3**	1.270	3.590	-1.497	0.002	0.038	0.929	3.594	-1.952	0.001	0.012	0.929	1.273	-0.454	0.036	0.684
	**YTHDC1**	0.950	0.500	0.913	0.000	0.001	1.106	0.504	1.134	0.001	0.015	1.106	0.949	0.221	0.034	0.646
	**YTHDC2**	1.260	0.380	1.730	0.000	0.001	1.211	0.381	1.668	0.004	0.076	1.211	1.264	-0.062	0.477	1.000
	**YTHDF1**	14.000	16.900	-0.278	0.002	0.038	24.891	16.944	0.555	0.010	0.190	24.891	13.976	0.833	0.006	0.114
	**YTHDF2**	1.530	3.470	-1.179	0.004	0.076	1.666	3.465	-1.056	0.005	0.095	1.666	1.530	0.123	0.010	0.190
	**YTHDF3**	1.040	0.960	0.116	0.241	1.000	0.980	0.958	0.033	0.454	1.000	0.980	1.038	-0.083	0.358	1.000
**ERASERS**	**ALKBH5**	0.170	0.280	-0.744	0.195	1.000	0.296	0.283	0.065	0.879	1.000	0.296	0.169	0.809	0.135	1.000
	**FTO**	14.300	6.360	1.169	0.009	0.171	3.786	6.358	-0.748	0.005	0.095	3.786	14.299	-1.917	0.003	0.057
